# Comparative epidemiology of outbreaks caused by SARS-CoV-2 Delta and Omicron variants in China

**DOI:** 10.1017/S0950268824000360

**Published:** 2024-03-19

**Authors:** Liping Peng, Xiaotong Huang, Can Wang, Hualei Xin, Benjamin J. Cowling, Peng Wu, Tim K. Tsang

**Affiliations:** 1 WHO Collaborating Centre for Infectious Disease Epidemiology and Control, School of Public Health, Li Ka Shing Faculty of Medicine, The University of Hong Kong, Hong Kong, China; 2 Laboratory of Data Discovery for Health Limited, Hong Kong Science and Technology Park, New Territories, Hong Kong, China

**Keywords:** COVID-19, Delta, Omicron, SARS-CoV-2, transmission

## Abstract

From 2020 to December 2022, China implemented strict measures to contain the spread of severe acute respiratory syndrome coronavirus 2. However, despite these efforts, sustained outbreaks of the Omicron variants occurred in 2022. We extracted COVID-19 case numbers from May 2021 to October 2022 to identify outbreaks of the Delta and Omicron variants in all provinces of mainland China. We found that omicron outbreaks were more frequent (4.3 vs. 1.6 outbreaks per month) and longer-lasting (mean duration: 13 vs. 4 weeks per outbreak) than Delta outbreaks, resulting in a total of 865,100 cases, of which 85% were asymptomatic. Despite the average Government Response Index being 12% higher (95% confidence interval (CI): 9%, 15%) in Omicron outbreaks, the average daily effective reproduction number (*R_t_*) was 0.45 higher (95% CI: 0.38, 0.52, *p* < 0.001) than in Delta outbreaks. Omicron outbreaks were suppressed in 32 days on average (95% CI: 26, 39), which was substantially longer than Delta outbreaks (14 days; 95% CI: 11, 19; *p* = 0.004). We concluded that control measures effective against Delta could not contain Omicron outbreaks in China. This highlights the need for continuous evaluation of new variants’ epidemiology to inform COVID-19 response decisions.

## Introduction

By the end of 2022, there were over 650 million confirmed COVID-19 cases and 6 million fatalities worldwide due to severe acute respiratory syndrome coronavirus 2 (SARS-CoV-2) infection [1]. SARS-CoV-2 has evolved and emerged into multiple variants, including Alpha, Beta, Delta [2], and Omicron [3]. The higher intrinsic transmissibility of Omicron subvariants poses a great challenge for control [4]. Although Omicron appears to cause milder infections in populations with high vaccination rates and/or prior infections [5], it retains a similar level of inherent severity to earlier variants in groups that have not been vaccinated or previously infected [6].

Prior to the global spread of Omicron in late 2021, approximately 110,000 COVID-19 cases were confirmed in China, with a rate of 7.7 cases per 100,000. These cases were primarily identified during the initial outbreak in Wuhan in early 2020. However, since the first detected importation of Omicron on 9 December 2021, in mainland China [7], multiple outbreaks have occurred in different areas. At its peak, there were daily recorded highs of 39,000 cases, and a total of 1.73 million cases were identified before the number of cases dropped to a national low of 14 daily cases on 25 June 2022. However, the number of cases could not reach zero and began to rise again. Despite the higher transmissibility of Omicron subvariants, mainland China has maintained the local elimination strategy called ‘Dynamic Zero-COVID’ during the summer 2022. This strategy aims to achieve daily zero local cases as often as possible. To control the Omicron outbreaks, stringent control measures have been implemented, including on-arrival polymerase chain reaction (PCR) test, hotel quarantine, mobile application-based contact tracing, risk-based routine mass PCR screening, and isolation of confirmed cases in hospitals or purpose-built facilities [8].

The transmission dynamics of outbreaks caused by the Delta and Omicron variants can be influenced by multiple factors. These factors include the effectiveness of control measures implemented during outbreaks, the level of vaccine coverage, and specific characteristics of the variants, such as the proportion of asymptomatic cases. In this study, we analysed and compared the epidemiological features of outbreaks in China caused by Delta in 2021 and Omicron in the summer 2022. We estimated the time-varying transmissibility and examined the temporal changes in case number, mobility, and government responses during these outbreaks. The objective of our study is to gain a deeper understanding of the epidemiological characteristics of these variants, with the aim of providing scientific evidence to support decision-making in response to the COVID-19 pandemic.

## Methods

### Case data

We obtained case data from 1 May 2021 to 14 October 2022, from the daily notification of COVID-19 on the National Health Commission of the People’s Republic of China (http://www.nhc.gov.cn/xcs/yqtb/list_gzbd.shtml). In China, cases were classified as follows [9]: (1) ‘confirmed cases’, referring to laboratory-confirmed cases presenting COVID-19-related symptoms (fever, dry cough, malaise, sore throat, loss of taste or smell, diarrhoea, etc.) and/or with radiographic evidence of lung infection and referred to as ‘symptomatic cases’ hereafter (Supplementary Table S1); (2) ‘asymptomatic cases’, referring to infections with laboratory-confirmed SARS-CoV-2 infection but without COVID-19-related symptoms [10]; (3) ‘cases converted from asymptomatic to symptomatic cases’ (pre-symptomatic cases), referring to previously asymptomatic cases who developed COVID-19-relevant symptoms and showed lung infiltrate in computed tomography (CT) scan; and (4) ‘imported cases’, referring to cases infected overseas who can be confirmed cases or asymptomatic cases.

In mainland China, all imported cases were detected during customs inspection and were promptly isolated in designated facilities. We excluded these imported cases from our analysis, with our primary focus being on local cases. Almost all local cases were found to have been infected by other local cases, except for a few cases that may have been infected by an imported case during the early stages of an outbreak. To determine the daily total number of cases, we combined the daily count of new symptomatic and asymptomatic cases and then subtracted the cases that were previously asymptomatic but had developed symptoms on that day.

### Mobility data

The daily migration data were sourced from the publicly available Baidu mobility big data (http://qianxi.baidu.com/). We collected Baidu mobility data for 358 prefecture-level cities, excluding Hong Kong, Macau, and Taiwan. The collected data encompassed information on both inter-city inflow and inter-city outflow. These data were aggregated into 31 province-level daily migration indices (inter-provincial inflow and inter-provincial outflow). To avoid weekly fluctuations induced by the work–leisure shift, the daily migration index was smoothed using a moving average over a 7-day window.

### Data about government response

The Oxford COVID-19 Government Response Tracker (OxCGRT, https://github.com/OxCGRT/covid-policy-tracker) collected publicly available information on various indicators of government responses to COVID-19. This information was aggregated into systematic indices [11]. We extracted three indices from this tracker: the Stringency Index (SI), capturing the changes in the school and workplace closure, containment measures, and public information campaigns; the Containment and Health Index (CHI), capturing the information in SI and the change in health policy; and the Government Response Index (GRI), a holistic measure of all information included in the above two indices and the change in economic support measures (Supplementary Table S4). A higher index score indicated that the government had implemented stricter policies in response to COVID-19.

### Definition of outbreaks

An outbreak was defined as a sudden increase in COVID-19 cases in one province with a maximum daily case number of 20 or more [12]. The start of an outbreak was defined as the date on which the first case, whether symptomatic or asymptomatic, was identified. On the other hand, the end of an outbreak was determined when there were no new cases reported for 7 consecutive days. We used the above definition to identify outbreaks in each province. Due to data availability, our analysis only focused on Delta outbreaks in 2021 and Omicron outbreaks occurred in March–June 2022. Outbreaks that were reported to have co-circulation of Delta and Omicron variants based on the government announcements were excluded from the study [13].

### Data visualization of case number, mobility, and government response

Heat maps were generated to depict temporal changes in case numbers, mobility, and government responses by province. We normalized the government response indices (SI, CHI, and GRI) and population mobility for each province by using the minimum value observed during the study period. Mobility data on National Day (1 October 2021 to 7 October 2021) and Chinese New Year (31 January 2022 to 6 February 2022) were excluded due to the exceptionally high mobility in these periods. Additionally, Pearson’s correlations were conducted to test the relationships between inter-provincial inflow, inter-provincial outflow, SI, CHI, and GRI at the province level.

### Estimation of time-varying effective reproduction number (R_t_)

To account for the potential of pre-symptomatic transmission for SARS-CoV-2 viruses [14], we reconstructed the epidemic curve by date of infection to estimate *R_t_.* The epidemic curve by infection date was reconstructed based on daily case numbers by report date using a deconvolution approach [15], allowing for the delay from infection to report (Supplementary Appendix, p. 2). Then, we estimated the *R_t_* based on the Poisson framework developed by Cori et al. [16] with daily confirmed case numbers and daily symptomatic case numbers*_._* The estimation of *R_t_* was implemented by the EpiEstim package in R [16]. We denoted the *R_t_* estimated based on symptomatic cases as 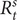 . The estimated *R_t_* and 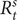 were compared by using a generalized estimation equation (GEE) with adjustments for province. We also calculated the duration of an outbreak and identified the number of days when *R_t_* and 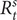 values first dropped below 1. When the *R_t_* / 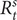 value dropped below 1, it indicated that an outbreak was under control. Specifically, we defined this point as the date when *R_t_* / 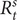 first decreased below 1 after reaching the peak of cases and continued to stay below 1 for a minimum of 7 consecutive days. The estimated daily *R_t_* in Delta and Omicron outbreaks was compared by GEE.

The confidence interval (CI) for the difference in the daily value of *R_t_* and the difference in the number of days for *R_t_* to drop to <1 were estimated using Bootstrap with 1,000 replications. The significance level for all tests was set at *p* < 0.05. To determine whether our results for the province level were also the same as for the city level, we extracted data on cases and mobility for 43 cities. These cities were selected based on three criteria: (1) 15 cities with Delta outbreaks, (2) the top 25 cities with the highest number of symptomatic cases in Omicron outbreaks, and (3) four additional cities were included to ensure that there was at least one city for each province with Omicron outbreaks in the province-level analysis. We repeated the above-mentioned analyses at the city level. Data scraping and visualizations were conducted using Python 3.8.6 (Python Software Foundation, Wilmington, DE), while data analysis was performed using R version 3.6.3 (R Foundation for Statistical Computing, Vienna, Austria).

## Results

**Figure 1.**
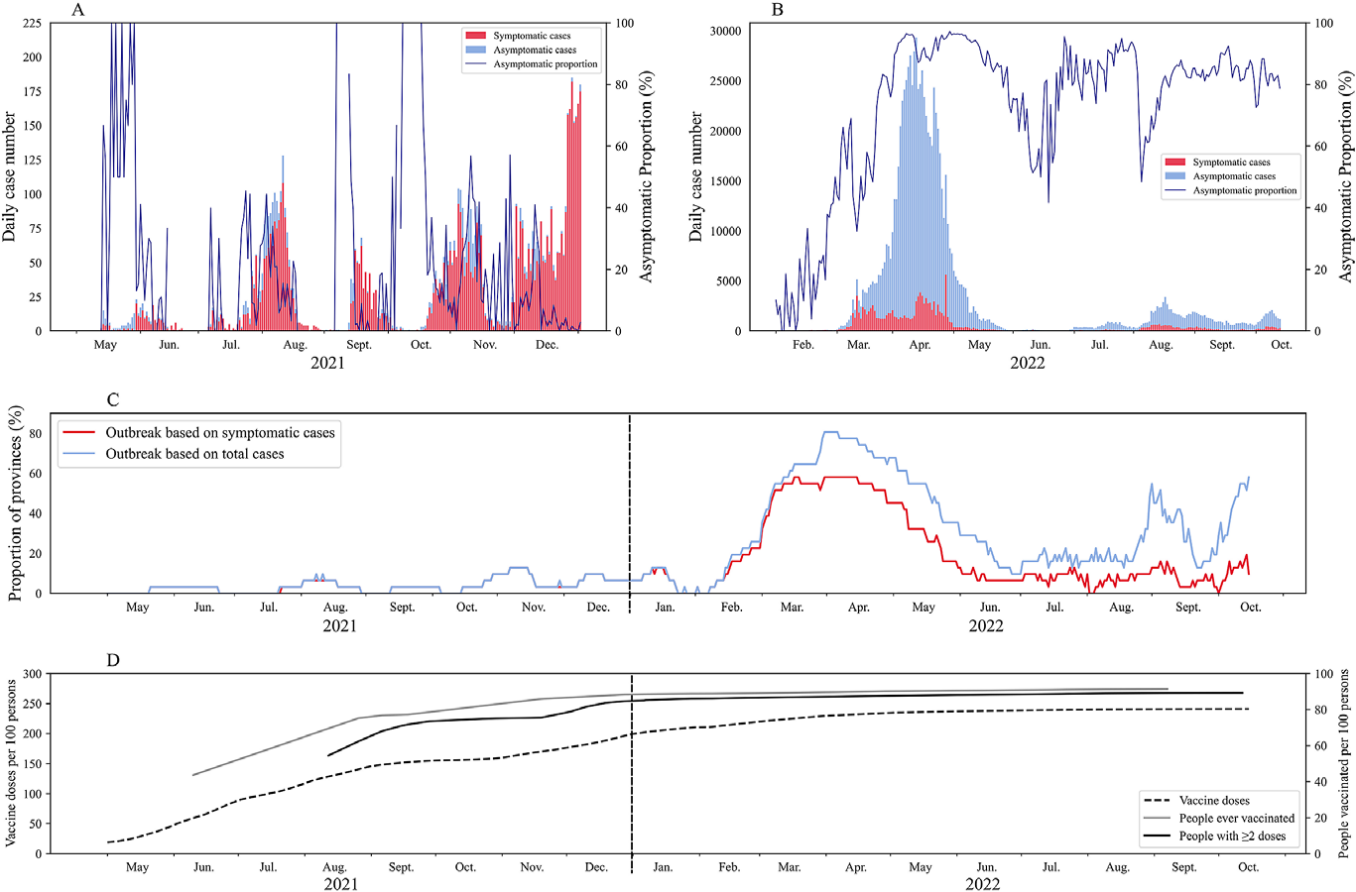
Illustration of COVID-19 epidemics in mainland China. (a,b) Symptomatic and asymptomatic COVID-19 local cases during May–December 2021 (Delta variants dominated) and during February–October 2022 (Omicron variants dominated). The solid blue line indicates the proportion of asymptomatic cases to the number of total cases on the day. (c) Proportion of province in outbreak according to symptomatic cases and total cases in mainland China during May 2021–October 2022. (d) Total vaccination dose, people received at least one dose, and ≥ 2 doses (full vaccination) per hundred persons in mainland China from May 2021 to October 2022.

In China, sporadic Delta outbreaks were reported in 10 provinces between May and December 2021 (Figure 1a). In total, there were 6,200 cases confirmed nationwide with over 90% being symptomatic and around 180 cases were identified daily during the peak of the outbreaks. During the 8 months in 2021, <10% (2/31) of the provinces were experiencing an outbreak (Figure 1), with only 1.6 outbreaks per month. In 2022, Omicron subvariants caused numerous outbreaks in 26 provinces, leading to 865,100 cases in total and 729,800 (84%) asymptomatic cases. The daily case numbers at the peak were close to 28,000. During the first wave (from March to June 2022), over 30% of the provinces were experiencing an outbreak on any given day (Figure 1), with an average of 4.3 outbreaks per month. The average proportion of asymptomatic cases was 85% across the 26 provinces, and this proportion exceeded 90% in some provinces, such as Shanghai and Hebei.

### Temporal changes in case number, mobility, and government response

We estimated that inter-provincial inflow and outflow were highly correlated, and also, the GRI, SI, and CHI were highly correlated (Supplementary Table S5). Therefore, we used GRI to indicate the intensity of government response and used inter-provincial inflow to indicate mobility. Except for five provinces, there was a negative correlation between mobility and government response in most provinces.

**Figure 2.**
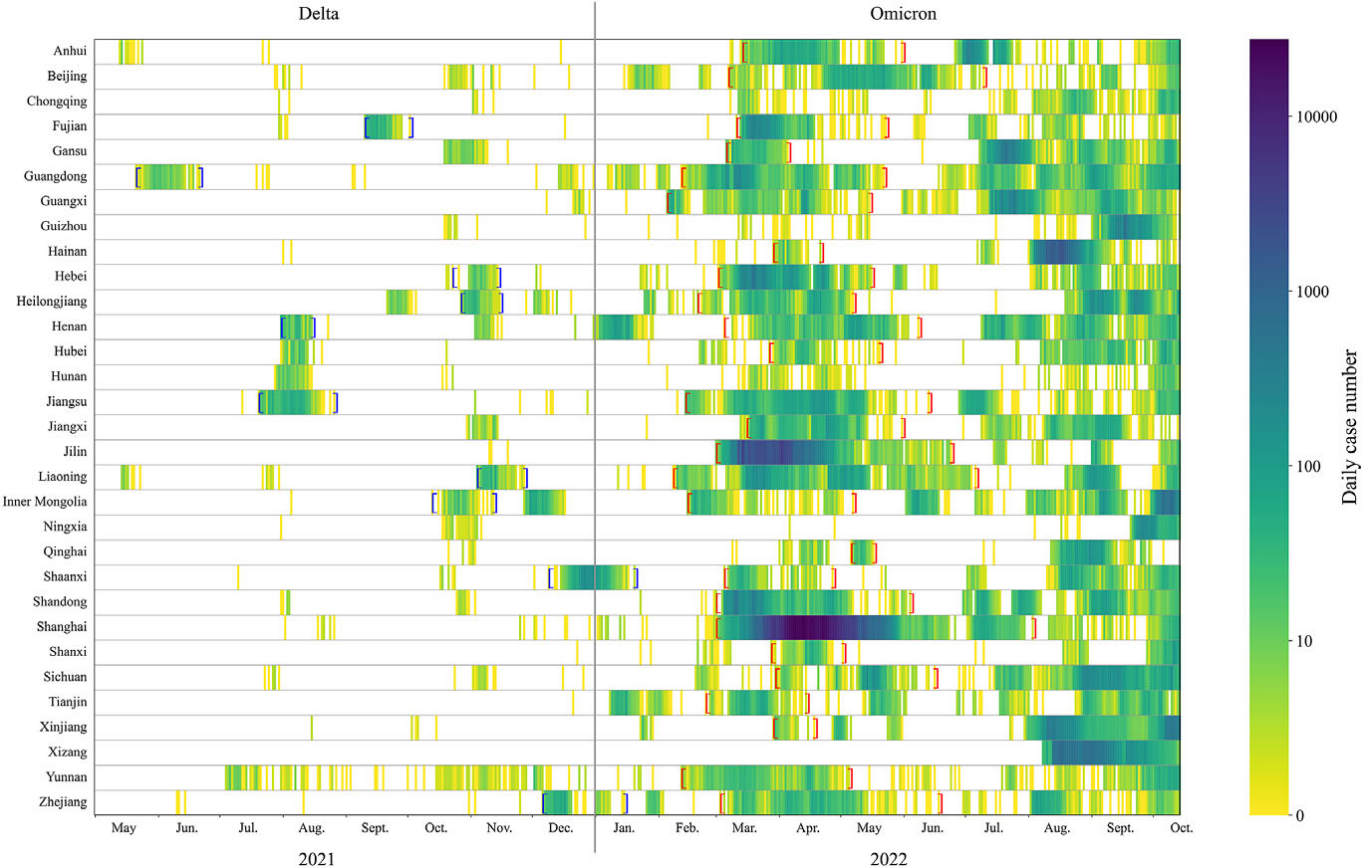
Heat map of case numbers by province during May 2021–October 2022. Blue brackets indicate the period of Delta outbreaks. Red brackets indicate the period of Omicron outbreaks.

The Omicron outbreaks in 2022 affected more provinces and resulted in a much greater number of cases than the Delta outbreaks in 2021 (Figure 2 and Supplementary Figure S2). Among 31 provinces, on average there were 204 days (91%) without any local cases reported from late May to December 2021, when the Delta variant was circulating (in total, 224 days). This is significantly higher than the 118 (46%) days when Omicron subvariants were circulating from February to October 2022 (a total of 254 days).

**Figure 3.**
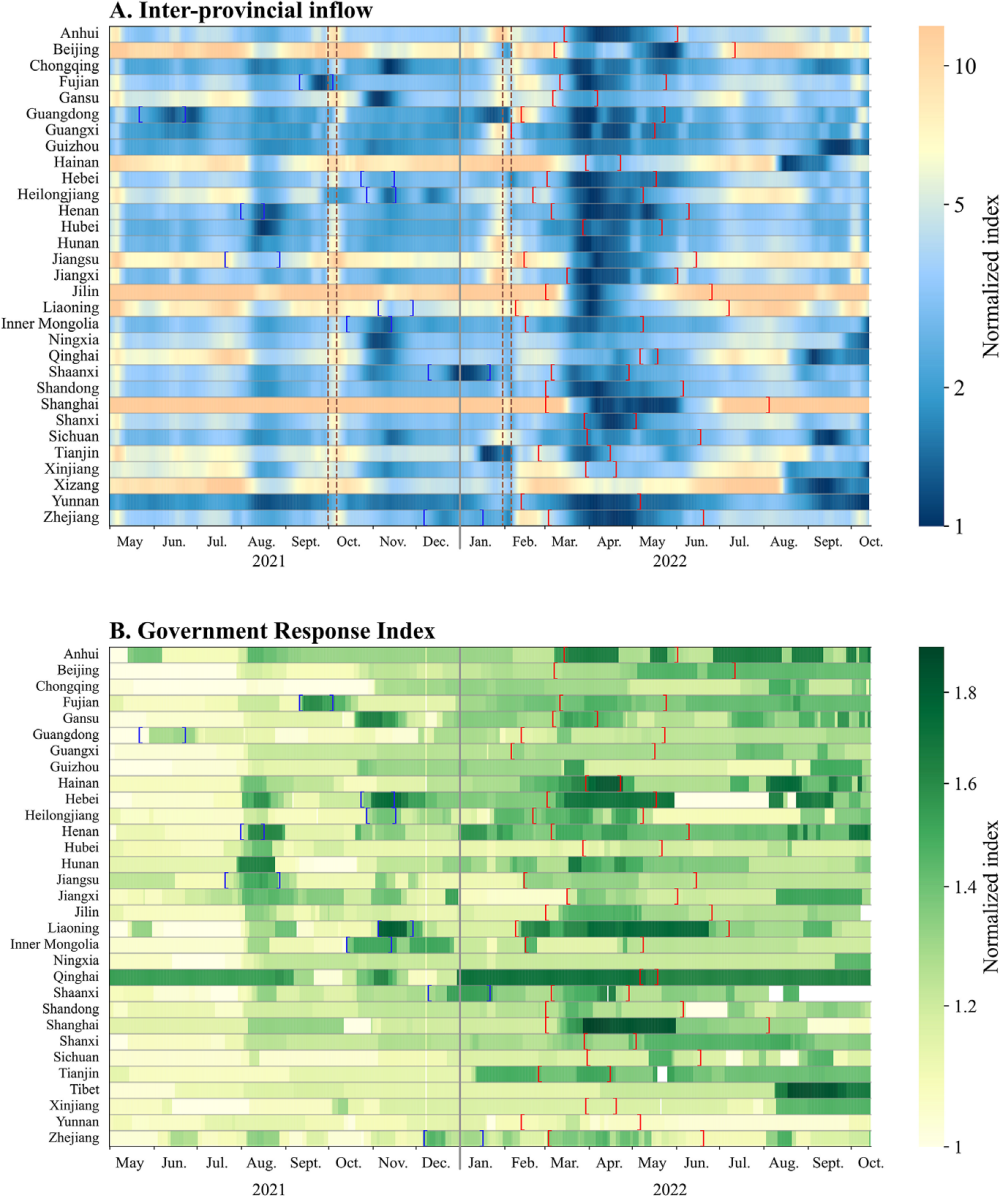
Heat map of changes in inter-provincial inflow and Government Response Index by province during May 2021–October 2022. Colour bars represent changes in the normalized index. Blue brackets indicate the period of Delta outbreaks. Red brackets indicate the period of Omicron outbreaks. Brown dashed lines indicate the period of National Day and Chinese New Year.

Some provinces tightened control measures in response to the Delta outbreaks in August and November 2021. In 2022, most provinces applied stricter measures for a longer period to contain the spread of Omicron. These measures resulted in a substantial decline in population mobility (Figure 3), particularly during mid-March to May 2022. During this period, almost all provinces observed a massive decrease in mobility.

Across all provinces, the average daily mobility (proxied by inter-provincial inflow) was 5% (95% CI: −2%, 12%) lower during the period when Omicron was circulating compared to the period with Delta. In city-level analysis, the average daily mobility was 7% (95% CI: 2%, 11%) lower during the period when Omicron was circulating compared to the period with Delta (Supplementary Figure S3). The same change was observed when mobility was proxied by inter-provincial/city outflow.

The average daily GRI, indicating the intensity of control measures, was 12% (95% CI: 9%, 15%) higher for Omicron period than for Delta period in all provinces. Among 10 provinces where outbreaks caused by both Delta and Omicron were reported, the average minimum inter-provincial inflow and outflow during Omicron outbreaks were 28% (95% CI: 10%, 46%) and 33% (95% CI: 14%, 48%) lower, respectively, than the time period when Delta outbreaks occurred. In terms of the intensity of control measures, the highest value of GRI during Omicron outbreaks was on average 4.5% (95% CI: 0.2%, 9.3%) lower than during Delta outbreaks, while 5 out of the 10 provinces applied more intensive control measures to respond to the Omicron outbreaks (Supplementary Figure S4).

### Time-varying effective reproduction number

**Figure 4.**
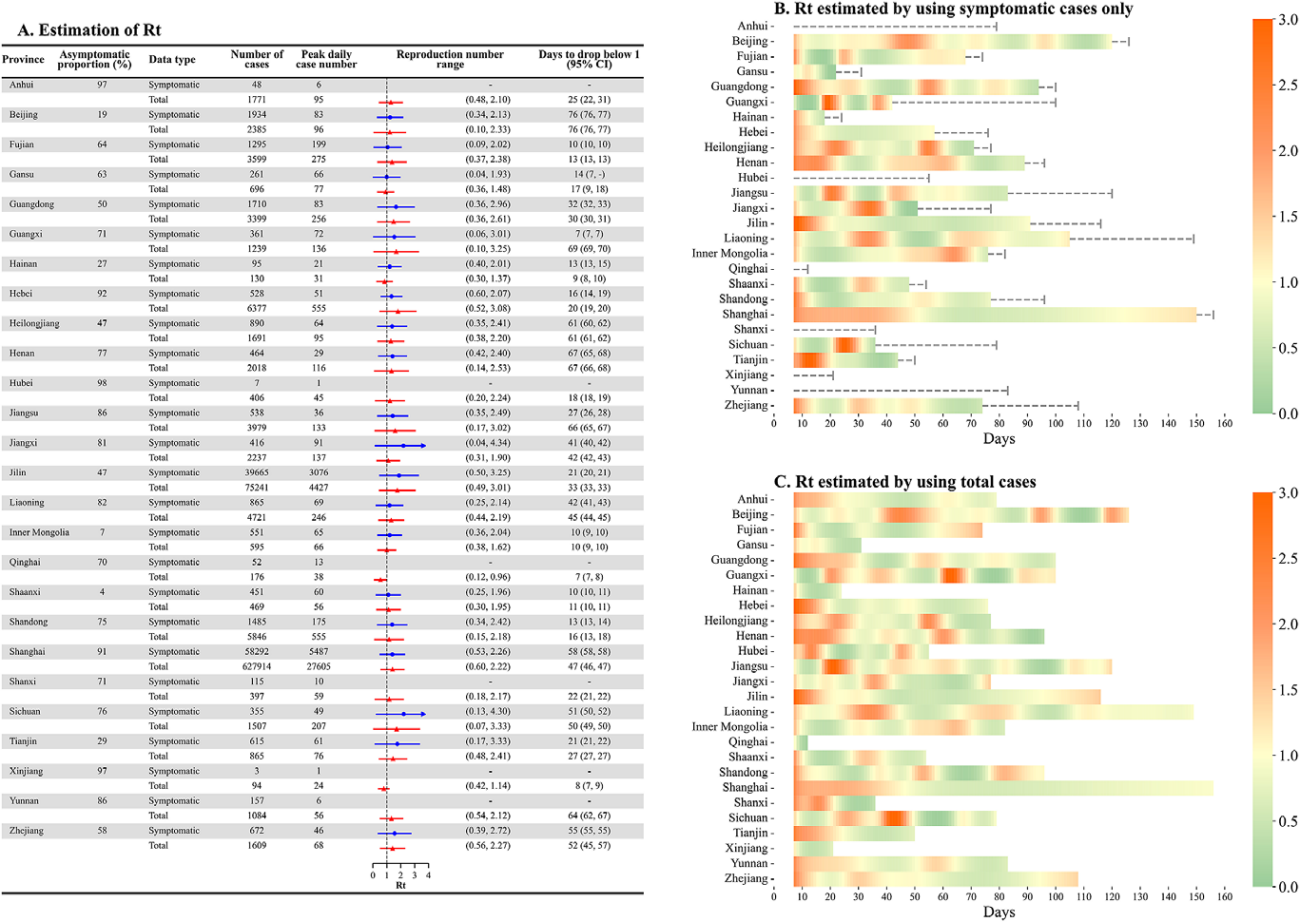
Estimation of time-varying effective reproduction number using daily reported symptomatic and total cases. (a) Epidemic description and estimated reproduction numbers by province. (b,c) Time-varying reproduction number; the *x*-axis indicates the time of outbreak progressed. The dashed line in panel (c) indicates the time difference in the end of an outbreak estimated using symptomatic cases and the total cases.

We estimated the *R_t_* of COVID-19 outbreaks for each of the provinces and cities included in the analysis (Supplementary Figures S5–S8). The estimated *R_t_* for Omicron increased since the outbreak began to nearly 3, fluctuated around 1 for 3–9 weeks, and finally went down to less than 0.5 indicating that the outbreak was under control (Figure 4). Province-level paired analyses for *R_t_* and 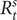 comparisons were conducted on 20 out of 26 provinces with Omicron outbreaks, as the excluded six provinces did not meet the outbreak definition based on symptomatic cases only. The estimated *R_t_* based on all cases was significantly higher and lower than the estimate of 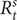 from symptomatic cases among three and four provinces after adjusting for multiple testing, respectively (Supplementary Table S6), among 20 provinces. Among 20 provinces with outbreaks, on average it took 32 days (range: 9–52) for the estimated *R_t_* to drop <1, with 12 (60%) provinces having a delay of 30 days or longer. There is no difference in the estimated number of days for *R_t_* and 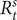 to drop <1 (*p* = 0.17).

### Comparison between Delta and Omicron outbreaks

Among 10 provinces that had Delta outbreaks in 2021 and Omicron outbreaks in 2022, the median number of cases reported in each Omicron outbreak was 2,641 (95% CI: 1603, 3,727), higher than in Delta outbreaks (mean: 542, 95% CI: 300, 871; *p* = 0.01). The peak daily number of cases in Omicron outbreaks (mean: 176, 95% CI: 102, 274) appeared to be higher than that observed in Delta outbreaks (mean: 64, 95% CI: 43, 94, *p* = 0.04). The duration of Delta outbreaks was 3.9 weeks (95% CI: 3.2, 4.6), which was shorter than that of Omicron outbreaks (13.1 weeks; 95% CI: 11.1, 15.3). The city-level analysis also suggested a significantly higher peak number of cases in and longer duration of Omicron outbreaks, compared with Delta outbreaks (Supplementary Appendix, p. 4).

**Figure 5.**
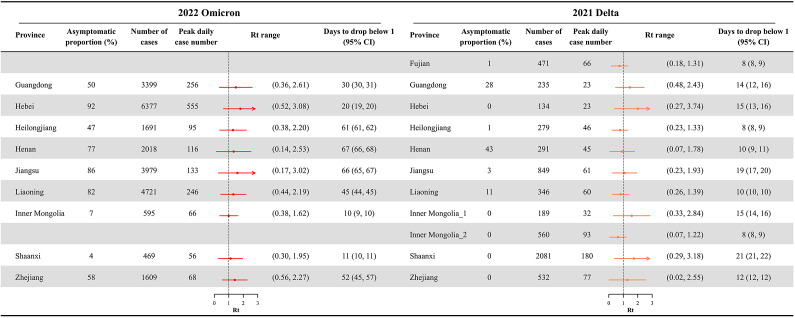
Comparison of major provincial outbreaks in 2021 and 2022. Total number of cases, peak daily case number, range of time-varying effective reproduction number (*R_t_*), and days for *R_t_* to drop below 1 are shown. Provinces were selected based on having outbreaks in both 2021 and 2022, with Inner Mongolia having two outbreaks in 2021. The *R_t_* was estimated based on all cases.

The average daily value of *R_t_* in Omicron outbreaks was 0.45 (95% CI: 0.38, 0.52, *p* < 0.001) higher than the estimates for Delta outbreaks. The number of days for *R_t_* to drop to <1 for Omicron outbreaks was 32 days (95% CI: 26, 39), much longer than for Delta outbreaks (14 days; 95% CI: 11, 19; *p* = 0.004). In addition, the proportion of asymptomatic cases increased markedly from 7.9% (95% CI: 0.7%–17.2%) during Delta outbreaks to 52.2% (95% CI: 31.1%–70.2%) during Omicron outbreaks in these 10 provinces (Figure 5). In the city-level analysis, we also observed significantly higher daily values of *R_t_*, longer duration for *R_t_* to drop to <1, and higher proportion of asymptomatic cases in Omicron outbreaks, compared with Delta outbreaks (Supplementary Figure S9).

## Discussion

In this study, we described and compared the epidemiological characteristics of outbreaks, caused by the Delta variant in 2021 and Omicron subvariants in summer 2022 in mainland China. We observed that Omicron subvariants spread more widely than Delta subvariants to all provinces in China, resulting in substantially higher case numbers in each outbreak and prolonged outbreaks [17]. The Omicron outbreaks in summer 2022 could not be controlled as effectively as for Delta [18] even with more intensive control measures corresponding to more substantial reductions in population mobility and higher vaccine coverage.

Our estimation revealed that the daily *R_t_* in Omicron outbreaks was significantly higher, and the number of days for *R_t_* to first drop <1 was also higher, compared to Delta outbreaks. These results were consistent with the much higher intrinsic transmissibility of Omicron compared to Delta, despite more stringent control measures being applied in Omicron outbreaks compared to Delta outbreaks. Also, Omicron spread to 26 out of 31 provinces as of October 2022 with a much higher case toll, compared to only nine provinces affected by Delta. This suggested that the inbound and domestic travel measures, as well as conventional contact tracing, case finding, and isolation, might not be sufficient to control Omicron transmission effectively compared to Delta transmission. This is due to the higher intrinsic transmissibility of Omicron variants and the milder presentations in infected individuals, particularly among those who have been vaccinated. As a result, infections could be more challenging to be detected [5, 19].

Consequently, our analysis based on the data from Omicron outbreaks in summer 2022 could already reveal that containment of Omicron subvariants may not be sustainable. Even with more frequent use of more stringent containment measures with substantial social disruption and economic costs [20], it never reached zero case again that we had observed after Delta outbreaks [18]. The occurrence of outbreaks in winter 2022 in almost all provinces in China served as a demonstration. Furthermore, repeated use of mobility restrictions may reduce their effectiveness due to pandemic fatigue [21].

In mainland China, definitions of confirmed COVID-19 cases have evolved since SARS-CoV-2 emerged [22]. The stricter definition for ‘asymptomatic cases’ in China, which involves the lack of evidence of lung infiltrates in CT scan in addition to the absence of relevant symptoms and signs [10], perhaps could have reduced the reported proportion of symptomatic cases in China if compared with the estimated proportions from other countries based purely on the presence or absence of symptoms for the same type of variant, although this would also depend on the exact definition of asymptomatic cases elsewhere [23]. The commonly used database only used the symptomatic case numbers for China that may also lead to substantial underestimation of pandemic severity in China [24]. The proportion of asymptomatic and mild infections in Omicron-infected individuals [25] was large, likely linked to the high vaccination coverage achieved in China. It may also be attributed to the large-scale PCR testing, which can identify more asymptomatic cases. Therefore, especially for Omicron outbreaks, estimating *R_t_* based on symptomatic cases only, as in most previous studies, may lead to bias that may jeopardize their ability to inform control policy.

We acknowledge several limitations in our analysis. First, increases in daily cases in a province might not necessarily result from a single outbreak but could reflect multiple co-incident outbreaks. We conducted an additional analysis based on city-level data, and the conclusions from province-level and city-level analyses were the same. Second, our study used province-level mobility data, but containment measures were often implemented at a smaller scale such as city or district. The province-level mobility data were likely to underestimate the changes in city- or district-level population movement in response to an outbreak, but this would not impact differently on the assessment of Delta and Omicron outbreaks. Third, we did not consider the impact of imported cases in our analysis. Nevertheless, given the strong border control measures, the transmissibility of imported cases would be close to zero and would have minimal impact on the estimate of the *R_t_* in local cases [26, 27]. Finally, when estimating *R_t_* we assumed that the delay from reporting was the same throughout both Delta and Omicron outbreaks. While we cannot rule out there could be change, China has consistently adhered to a zero-COVID policy and implemented strict monitoring, testing, and case reporting measures. Case reporting and government response have also been prompt. Extensive and mass testing was initiated when there was a case in a city, supporting that the difference in report delay would not be significant.

In conclusion, our study demonstrated that previously effective measures in control of outbreaks of earlier variants of SARS-CoV-2 could not contain Omicron outbreaks even with a more stringent implementation of similar measures for a longer period. Ongoing epidemiological evaluation would remain critical, particularly in assessing the transmissibility and severity of new virus variants, in order to better develop appropriate pharmaceutical and non-pharmaceutical interventions, thereby minimizing the related health and social costs.

## Supporting information

Peng et al. supplementary material 1 Peng et al. supplementary material

Peng et al. supplementary material 2 Peng et al. supplementary material

Peng et al. supplementary material 3 Peng et al. supplementary material

Peng et al. supplementary material 4 Peng et al. supplementary material

Peng et al. supplementary material 5 Peng et al. supplementary material

Peng et al. supplementary material 6 Peng et al. supplementary material

Peng et al. supplementary material 7 Peng et al. supplementary material

Peng et al. supplementary material 8 Peng et al. supplementary material

Peng et al. supplementary material 9 Peng et al. supplementary material

Peng et al. supplementary material 10 Peng et al. supplementary material

## Data Availability

The data sets generated and analysed during the current study are available from publicly available sources as follows: (1) COVID-19 case data come from the National Health Commission of the People’s Republic of China (http://www.nhc.gov.cn/xcs/yqtb/list_gzbd.shtml); (2) mobility data come from Baidu mobility big data (http://qianxi.baidu.com/); and (3) government response data come from the Oxford COVID-19 Government Response Tracker (OxCGRT, https://github.com/OxCGRT/covid-policy-tracker).

## Supplementary material

The supplementary material for this article can be found at https://doi.org/10.1017/S0950268824000360.
