# Supplementary material for: Comparative epidemiology of outbreaks caused by SARS-CoV-2 Delta and Omicron variants in China
Source: Epidemiol Infect. 2024 Mar 19;152:e43. doi: 10.1017/S0950268824000360 (PMC10964185; doi:10.1017/S0950268824000360)
Supplement: Peng et al. supplementary material 1 — Peng et al. supplementary material [file S0950268824000360sup001.docx]

**Supplementary Material**

**for ‘Comparative epidemiology of outbreaks caused by SARS-CoV-2 Delta and Omicron variants in China: an observational study’**

Contents

[1. Estimation of time-varying effective reproductive number 2](#_Toc153203753)

[1.1 Details of models 2](#_Toc153203754)

[1.2 Likelihood function 2](#_Toc153203755)

[1.3 Likelihood function 3](#_Toc153203756)

[2 Data analysis of COVID-19 outbreak in China 3](#_Toc153203757)

[2.1 Inference of epidemic curve by infection date 3](#_Toc153203758)

[2.2 Assumption on input parameter in data analysis 3](#_Toc153203759)

[3. City-level analysis of comparing Delta and Omicron outbreaks 4](#_Toc153203760)

[4. Vaccine coverage in outbreaks caused Delta and Omicron 5](#_Toc153203761)

# 1. Estimation of time-varying effective reproductive number

## 1.1 Details of models

We used the framework in Cori et al.[1] to estimate the R_t_. The model assumed transmission follows a Poisson process. Denote $w_{s}$ the probability distribution of the infectiousness profile since infection, the rate of individuals infected at time step t-s generates new infections in time step t was equal to $R_{t}w_{s}$, where $R_{t}$ was the instantaneous reproductive number at t. Also, the incidence at time t was Poisson distributed with mean $R_{t}\sum_{s=1}^{t} I_{t-s}w_{s}.$

Denote $Y(k)$ the number of new cases infected on day k. Then, we have:

$$Y\left( t \right)\sim Poisson\{R\left( t \right)\sum_{k=1}^{t-1} Y\left( k \right)w\left( t-k \right)\}$$

where $R\left( t \right)$ are the time-varying effective reproductive number at time t.

## 1.2 Likelihood function

To avoid bumpy estimates, smoothing method as in Cori et al. was applied. It was achieved by assuming that the transmissibility was constant over a time period $[t-\tau+1,t]$, where $\tau$ is the smoothing parameter. Hence likelihood at a day *t* was

$$P\left( Y\left( t \right),\ldots,Y\left( t-\tau+1 \right) | Y\left( 1 \right),\ldots Y\left( t-\tau\right) \right)=\prod_{s=t-\tau+1}^{t} \frac{\left( R^{\tau}\left( t \right)\phi\left( s \right) \right)^{Y\left( s \right)}e^{-R^{\tau}\left( t \right)\phi\left( s \right)}}{Y\left( s \right)!}$$

where $\phi\left( t \right)=\sum_{k=1}^{t-1} Y\left( k \right)w\left( t-k \right)$. The total likelihood was the product of individual likelihood at each time *t* in the observed data. The first $\tau-1$ days were excluded due to $\tau$-day smoothing.

## 1.3 Likelihood function

After obtaining the time series of case by infection date from deconvolution of time series of case by report date (details were in below), we use the EpiEstim[1] package to estimate the $R_{t}$.

# 2 Data analysis of COVID-19 outbreak in China

Since the time series of cases by infection dates could not be observed, we need to infer the epidemic curve by infection dates, to perform estimation of time-varying reproduction number. The prior for $R_{t}$ was Gamma(1,5) with mean and standard deviation equal to 5.

## 2.1 Inference of epidemic curve by infection date

We used deconvolution approach in Miller et al.[2] to obtain the epidemic curve by infection time from the epidemic curve by onset time, and a given distribution of delay from infection to report. This approach was implemented by the package ‘incidental’.

## 2.2 Assumption on input parameter in data analysis

For incubation period distribution, we use the estimate mean 3.2 days (SD 2.2) estimated from Backer et al. [3] for Omicron and mean 4.4 days (SD 2.5) for Delta.

Due to China's consistent adherence to strict monitoring, testing, and case reporting measures, it is worth noting that as of January 2021, China's daily capacity of nucleic acid testing reached 15 million samples,[4] and by April 2022, the capacity reached 51.65 million samples per day.[5] With such sufficient nucleic acid testing capacity, there is support for rapid case reporting. Consequently, for our analysis, we assumed a 50/50 split between 1-day delay and 2-day delay from onset to report, then we can construct the distribution for the delay from infection to report by convolution of incubation period distribution and the empirical distribution of delay from onset to report. This distribution would be used for deconvolution approach.

The infectiousness since infection $w_{t}$ was a generation time distribution, assumed to be Gamma distribution, with estimated mean 3.3 days and SD 2.4[3] for Omicron outbreaks, and mean 3.5 days (SD 2.8) for Delta outbreaks.

We take 7 days smoothing in the Poisson framework in Cori et al, to avoid unstable estimates for time-varying reproductive number.

# 3. City-level analysis of comparing Delta and Omicron outbreaks

In city-level analysis, the median number of cases reported in each Omicron outbreak were 3085 (95% CI: 833, 6480), higher than in Delta outbreaks (mean: 398, 95% CI: 224, 658; *p* = 0.013) (Figure S2). The peak daily number of cases in Omicron outbreaks (mean: 334, 95% CI: 113, 650) appeared to be higher than the observed in Delta outbreaks (mean: 52, 95% CI: 36, 76, *p* = 0.030). The duration of Delta outbreaks was 3.0 weeks (95% CI:2.4, 3.6), which were shorter than that of Omicron outbreaks (5.7 weeks; 95% CI: 4.7, 6.7, *p* < 0.001).

Among 8 cities that had Delta outbreaks in 2021 and Omicron outbreaks in 2022, the daily values of *R_t_* in Omicron outbreaks were 0.26 (95% CI: 0.14, 0.37, *p* < 0.001) higher than the estimates for Delta outbreaks. The number of days for *R_t_* to drop to <1 for Omicron outbreaks was 19 days (95% CI: 15, 24), longer than for Delta outbreaks (11 days; 95% CI: 9, 13; *p* = 0.004). In addition, the proportion of asymptomatic cases increased markedly from 10.9% (95% CI: 0.5%-17.8%) during Delta outbreaks to 57.5% (95% CI: 46.2%-66.8%) (Figure S14).

# 4. Vaccine coverage in outbreaks caused Delta and Omicron

Mass vaccination began in late December 2020 in China for high-risk groups and eligible adults, and was extended to the elderly in April 2021,[6] and further to children at age of 3-17 years in June 2021.[7] Among eligible people, the vaccine coverage of 1-dose surpassed 50% in August 2021.[8] By January 2022 before the Omicron outbreaks, the vaccine coverage of 1-dose and 2-dose were 90% and 87%, respectively, and in April 2022, the coverage of vaccination with booster doses reached 50%.[9] By November 2022, the 1-dose, 2-dose and 3-dose vaccine coverage were 93%, 90% and 58%.[10] There were 86% and 69% of the elderly aged ≥60y vaccinated with 2-doses and 3-dose respectively, but the 2-dose and 3-dose vaccine coverage were only 66% and 40% for the elderly aged ≥80y, respectively[10] (Table S2). To enhance the understanding of the use of vaccines in China, we have compiled information about vaccine brands used in the China (Table S3).

**SUPPLEMENTARY FIGURE LEGEND**

**Figure S1.** Epidemic curve of Omicron outbreaks in early 2022 on province-level. Blue lines indicate symptomatic cases, and red lines indicate asymptomatic cases.

**Figure S2**. Heat map of case number on city-level during May 2021– October 2022.

**Figure S3**. Heat map of changes in mobility on city-level during May 2021– October 2022. Color bars represented changes in the normalized index. Brown dashed lines indicate the period of National Day and Chinese New Year.

**Figure S4**. Radar chart of different containment and closure indicators (C1-C8) with maximum scores in provinces with both Delta and Omicron outbreaks.

**Figure S5**. Time-varying reproduction number of provinces during Omicron outbreak in early 2022. Blue lines indicate $R_{t}^{s}$ (estimated based on symptomatic cases), and red lines indicate *R_t_* (estimated based on symptomatic cases and asymptomatic cases).

**Figure S6**. Time-varying reproduction number of provinces during Delta outbreak.

**Figure S7**. Time-varying reproduction number of cities during Omicron outbreak in early 2022. Blue lines indicate $R_{t}^{s}$ (estimated based on symptomatic cases), and red lines indicate *R_t_* (estimated based on symptomatic cases and asymptomatic cases).

**Figure S8**. Time-varying reproduction number of cities during Delta outbreak.

**Figure S9**. Comparison of major city-level outbreaks in 2021 and 2022. Total numbers of cases, peak daily case number, range of time-varying effective reproduction number (*R_t_*), and days for Rt to drop below 1 are shown. The *R_t_* was estimated based on all cases.

**Table S1.** Definitions of suspected and confirmed cases of COVID-19 in nine versions of the National Guideline for Diagnosis and Treatment of the Novel Coronavirus Infection implemented in China in Delta and Omicron outbreaks.

| Version of guideline | Case definitions | Difference from the previous version | Date of issue | Date of online publication* |
| --- | --- | --- | --- | --- |
| 8 | Suspected cases:  Meet any of the epidemiological criteria and any two of the clinical criteria OR meet any two of the clinical criteria and positive SARS-CoV-2-specific IgM antibody in those not meeting any epidemiologic evidence OR meet all three clinical criteria in those not meeting any epidemiology criteria.  1.Epidemiological history  (1) Travel history or residence in a community with case reports within 14 days before the onset;  (2) History of contact with patients with COVID-19 or asymptomatic infection within 14 days of the onset;  (3) Contact with patients with fever or respiratory symptoms from communities with case reports within 14 days of the onset;  (4) Cluster cases (2 or more cases of fever and/or respiratory symptoms occurring in small areas such as homes, offices, school classes, etc.) within 14 days.  2. Clinical criteria  (1) Fever and/or respiratory symptoms consistent with COVID-19;  (2) Radiologic features compatible with COVID-19;  (3) Normal or decreased white cell count and lymphocyte count in the early stage.  Confirmed cases  Suspected cases who meeting any of the following criteria:  1. RT-PCR test positive for SARS-CoV-2;  2. Gene sequencing of the virus highly homologous to the known sequence of SARS-CoV-2;  3. Positive tests for SARS-CoV-2-specific IgM and IgG antibodies;  4. Seroconversion of SARS-CoV-2-specific IgG antibody, or 4-fold increase in IgG antibody titer in convalescent plasma than that in the acute phase. | 1. Broadened the contact history to any communities rather than within Wuhan/Hubei  2. Separated serology into positive tests and seroconversion | Aug 18, 2020 | September 7, 2020 on NHC website |
| 9 | Suspected cases:  Have any of the following epidemiological histories and meet any two clinical manifestations; if there is no clear epidemiological history, meet three of the clinical manifestations; or have any two of the clinical manifestations and the 2019‐nCoV‐specific IgM antibody is positive (not used for recently vaccinated persons).  1.Epidemiological history  (1) Travel or residence history in a community with case reports within 14 days before the onset;  (2) A history of contact with patients with 2019‐nCoV infection within 14 days of the onset;  (3) Contact with patients with fever or respiratory symptoms from communities with case reports within 14 days of the onset;  (4) Clustering disease (two or more cases of fever and/or respiratory symptoms occurring in small areas, such as homes, offices, and schools) within 14 days  2. Clinical manifestations  (1) Fever and/or respiratory symptoms and other above-mentioned clinical manifestations related to COVID‐19;  (2) Have imaging characteristics ofCOVID‐19;  (3) The total number of white blood cells is low or normal at the early stage, and lymphocyte count is low or normal.  Confirmed cases  Suspected cases with any one of the following etiological or serological evidence:  (1) A positive real‐time RT‐PCR detection of the2019‐nCoV nucleic acid;  (2) The 2019‐nCoV‐specific IgM and IgG antibody tests are positive for the unvaccinated person | 1. Restricted antibody positive confirmation to persons who are not vaccinated recently | Mar 15, 2022 | Mar 15, 2022 |

* Online publication refers to whether the document was available on the official websites of NHC and China CDC.

**Table S2.** The timeline of vaccine policy and vaccine coverage in China.

| Time | Event | Sources |
| --- | --- | --- |
| December 15, 2020 | High risk groups, including staffs engaged in import cold chain, port quarantine, ship piloting, aviation air crew, fresh market, public transportation, medical, disease control, and those who is going to work or study in medium- and high-risk countries, are eligible for vaccination | http://www.gov.cn/xinwen/gwylflkjz140/index.htm |
| December 30, 2020 | Adults (18-59) are eligible for vaccination | https://www.bbc.com/zhongwen/trad/science-55495307 |
| January 9, 2021 | >9.1 million doses (0.84 doses per 100 eligible person) are given | http://www.gov.cn/xinwen/gwylflkjz144/index.htm |
| March 1, 2021 | Coverage of at least 1 dose: 3.56% | https://www.guancha.cn/politics/2021_03_02_582714.shtml |
| April 1, 2021 | Elderly (60+) are eligible for vaccination | http://gdstc.gd.gov.cn/kjfyzl/gnwdt/content/post_3261369.html |
| Apr 21, 2021 | Vaccine coverage for healthcare worker: >80% | http://www.gov.cn/xinwen/gwylflkjz155/index.htm |
| April 28, 2021 | 235.976 million doses (21.87 doses per 100 eligible person) are given | http://www.nhc.gov.cn/xcs/yqjzqk/202104/6fa3e91fe96f424ab144c1f33fcafab9.shtml |
| May 10, 2021 | 416.938 million doses (38.64 doses per 100 eligible person) are given | http://www.nhc.gov.cn/xcs/yqjzqk/202105/2636a2e16c6c4fc89e2358bd3898b9ce.shtml |
| June, 2021 | Children (3-17) are eligible for vaccination. However the mass vaccination campaign for children for 3-11 began in late October 2021. | <http://wsjkw.hebei.gov.cn/html/zwyw/20211026/383388.html>  https://www.nytimes.com/2021/12/06/business/china-covid-vaccine-children.html?_ga=2.1079478.605070662.1669704296-1034177539.1669704296 |
| June 25, 2021 | Coverage of at least 1 dose: >40% | https://news.cctv.com/2021/06/25/ARTIoJoxDeNS9htZFjPdBPNz210625.shtml |
| August 12, 2021 | 1832.45 million doses (169.85 doses per 100 eligible person) are given  Coverage of at least 1 dose: >50% | http://www.nhc.gov.cn/xcs/fkdt/202108/6c77e46253b84b1f9cca2d5aa4a0f76b.shtml |
| August 26, 2021 | 2003.944 million doses (185.74 doses per 100 eligible person) are given  Coverage of at least 1 dose: >60% | http://m.news.cctv.com/2021/08/27/ARTI57PlijMcRRfQ7FwMbt6F210827.shtml |
| September 18, 2021 | Coverage of at least 2 doses: 78% | http://health.people.com.cn/n1/2021/0920/c14739-32231805.html |
| November 19, 2021 | Coverage of at least 1 dose: 86.9%  Coverage of at least 2 doses: 76.3%  Coverage of at least 3 doses: 4.66% | http://www.news.cn/politics/2021-11/20/c_1128083263.htm |
| December 6, 2021 | Coverage of at least 1 dose for age 3-11: 50% | https://www.nytimes.com/2021/12/06/business/china-covid-vaccine-children.html?_ga=2.1079478.605070662.1669704296-1034177539.1669704296 |
| December 28, 2021 | Coverage of at least 1 dose: 89.37%  Coverage of at least 2 doses: 85.64% | http://www.nhc.gov.cn/xcs/fkdt/202112/72c7929f82d541c89d846d8a67f35995.shtml |
| January 15, 2022 | Coverage of at least 1 dose: 89.63%  Coverage of at least 2 doses: 86.58% | http://www.nhc.gov.cn/xcs/fkdt/202201/956b9a4d566546d7966d5cf93641bd2a.shtml |
| March 31, 2022 | Coverage of at least 1 dose: 90.63%  Coverage of at least 2 doses: 88.11%  Coverage of at least 3 doses: 49.29 % | https://web.archive.org/web/20220401133412/http://news.china.com.cn/2022-04/01/content_78143598.html |
| April 5, 2022 | Coverage of at least 1 dose: 90.70%  Coverage of at least 2 doses: 88.18%  Coverage of at least 3 doses: 50.05 % | http://www.gov.cn/xinwen/gwylflkjz191/index.htm |
| April 28, 2022 | Coverage of at least 1 dose: 91.12%  Coverage of at least 2 doses: 88.62%  Coverage of at least 3 doses: 53.10%  Coverage of at least 1 dose for elderly (60+): 85.94%  Coverage of at least 2 doses for elderly (60+): 81.44%  Coverage of at least 3 doses for elderly (60+): 60.57% | http://www.nhc.gov.cn/xcs/fkdt/202204/34ce2f4566844cee9c0b2f0a5afbf6f7.shtml |
| July 20, 2022 | Coverage of at least 1 dose: 92.08%  Coverage of at least 2 doses: 89.68%  Coverage of at least 3 doses: 56.55%  Coverage of at least 1 dose for elderly (60+): 89.45%  Coverage of at least 2 doses for elderly (60+): 84.5%  Coverage of at least 3 doses for elderly (60+): 66.19% | http://www.nhc.gov.cn/xcs/fkdt/202207/10b358da7913436aabd12fc1cf6a8f5b.shtml |
| Aug 9, 2022 | Coverage of at least 1 dose: 92.31%  Coverage of at least 2 doses: 89.96%  Coverage of at least 3 doses: 56.9%  Coverage of at least 1 dose for elderly (60+): 90.44%  Coverage of at least 2 doses for elderly (60+): 85.63%  Coverage of at least 3 doses for elderly (60+): 66.76% | https://finance.sina.cn/2022-08-10/detail-imizirav7563774.d.html?from=wap |
| September 7, 2022 | Coverage of at least 1 dose: 92.40%  Coverage of at least 2 doses: 90.13%  Coverage of at least 3 doses: 57.15%  Coverage of at least 1 dose for elderly (60+): 90.57%  Coverage of at least 2 doses for elderly (60+): 86.14%  Coverage of at least 3 doses for elderly (60+): 67.06% | http://www.nhc.gov.cn/xcs/s3574/202209/6633cf47a8e640c1863c4c2858532957.shtml |
| September 28, 2022 | Coverage of at least 1 dose: 92.44%  Coverage of at least 2 doses: 90.18%  Coverage of at least 3 doses: 57.25%  Coverage of at least 1 dose for elderly (60+): 90.59%  Coverage of at least 2 doses for elderly (60+): 86.26%  Coverage of at least 3 doses for elderly (60+): 67.18% | http://www.nhc.gov.cn/xcs/s3574/202209/81ac71236c4f47cc8843f71dfb9b8aa1.shtml |
| October 12, 2022 | Coverage of at least 1 dose: 92.45%  Coverage of at least 2 doses: 90.2%  Coverage of at least 3 doses: 57.30%  Coverage of at least 1 dose for elderly (60+): 90.6%  Coverage of at least 2 doses for elderly (60+): 86.3%  Coverage of at least 3 doses for elderly (60+): 67.25% | http://www.nhc.gov.cn/xcs/s3574/202210/73d717e4fd82496b8075a6aa69e9d038.shtml |
| November 4, 2022 | Coverage of at least 1 dose: 92.48%  Coverage of at least 2 doses: 90.24%  Coverage of at least 3 doses: 57.40%  Coverage of at least 1 dose for elderly (60+): 90.62%  Coverage of at least 2 doses for elderly (60+): 86.35%  Coverage of at least 3 doses for elderly (60+): 68.45% | http://www.nhc.gov.cn/xcs/s3574/202211/39d316d555094db5b2a6a9eb97214a8b.shtml |
| November 11, 2022 | Coverage of at least 1 dose: 92.5%  Coverage of at least 2 doses: 90.26%  Coverage of at least 1 dose for elderly (60+): 90.65%  Coverage of at least 2 doses for elderly (60+): 86.38%  Coverage of at least 3 doses for elderly (60+): 68.19%  Coverage of at least 2 doses for elderly (80+): 65.7%  Coverage of at least 3 doses for elderly (80+): 40% | http://www.nhc.gov.cn/xcs/s3574/202211/b20c7ccccf4842af874a7781392311e5.shtml |
| November 28, 2022 | Coverage of at least 1 dose: 92.54%  Coverage of at least 2 doses: 90.28%  Coverage of at least 3 doses: 57.54%  Coverage of at least 1 dose for elderly (60+): 90.68%  Coverage of at least 2 doses for elderly (60+): 86.42%  Coverage of at least 3 doses for elderly (60+): 68.75%  Coverage of at least 1 dose for elderly (80+): 76.6%  Coverage of at least 3 doses for elderly (80+): 65.8%  Coverage of at least 3 doses for elderly (80+): 40.37% | https://china.caixin.com/2022-11-29/101971919.html |

**Table S3.** Vaccines approved for use in China[11]

| Vaccine | Type | Made by | Deployment |
| --- | --- | --- | --- |
| Sinopharm BIBP | Inactivated | Beijing Institute of Biological Products | December, 2020 |
| CoronaVac | Inactivated | Sinovac Biotech | February, 2021 |
| Sinopharm WIBP | Inactivated | Wuhan Institute of Biological Products | February, 2021 |
| KCONVAC | Inactivated | Kangtai Biological Products Co. Ltd. | June, 2021 |
| Covidful | Inactivated | Institute of Medical Biology, Chinese Academy of Medical Sciences | June, 2021 |
| Convidecia | Viral vector | CanSino | February, 2021 |
| ZIFIVAX | Protein Subunit | Zhifei Bio-tech | March, 2021 |
| Livzon | Protein Subunit | Livzon Mabpharm Inc | September, 2022 |
| CanSino Air | Viral vector | CanSino | October, 2022 |

**Table S4**. Summary of OxCGRT indicators


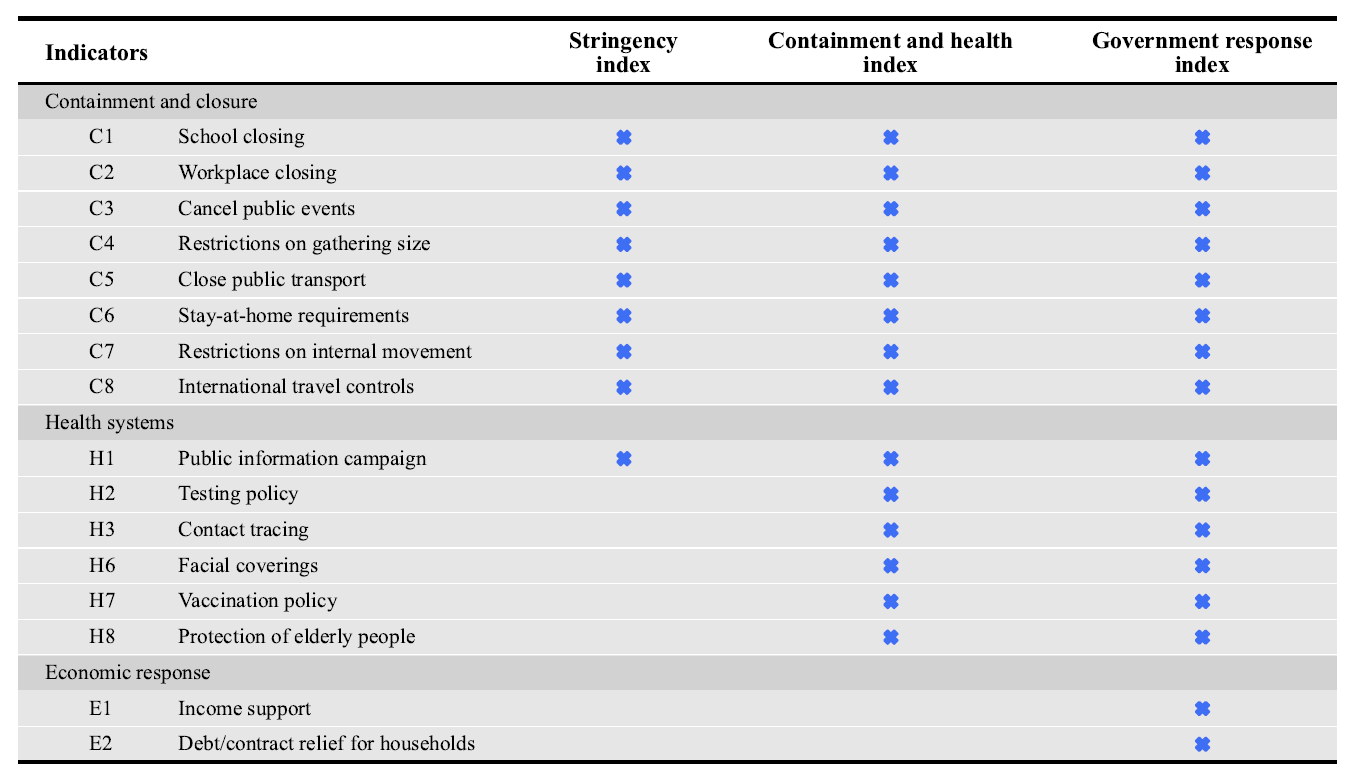


**Table S5**. Correlation of mobility and OxCGRT indices during outbreaks. M1 is for inter-provincial inflow, M2 is for inter-provincial outflow, S is for Strigency index, C is for Containment and Health Index, and G is for Government Response Index. *** *p* < 0.001, ** *p* < 0.01, * *p* < 0.05.


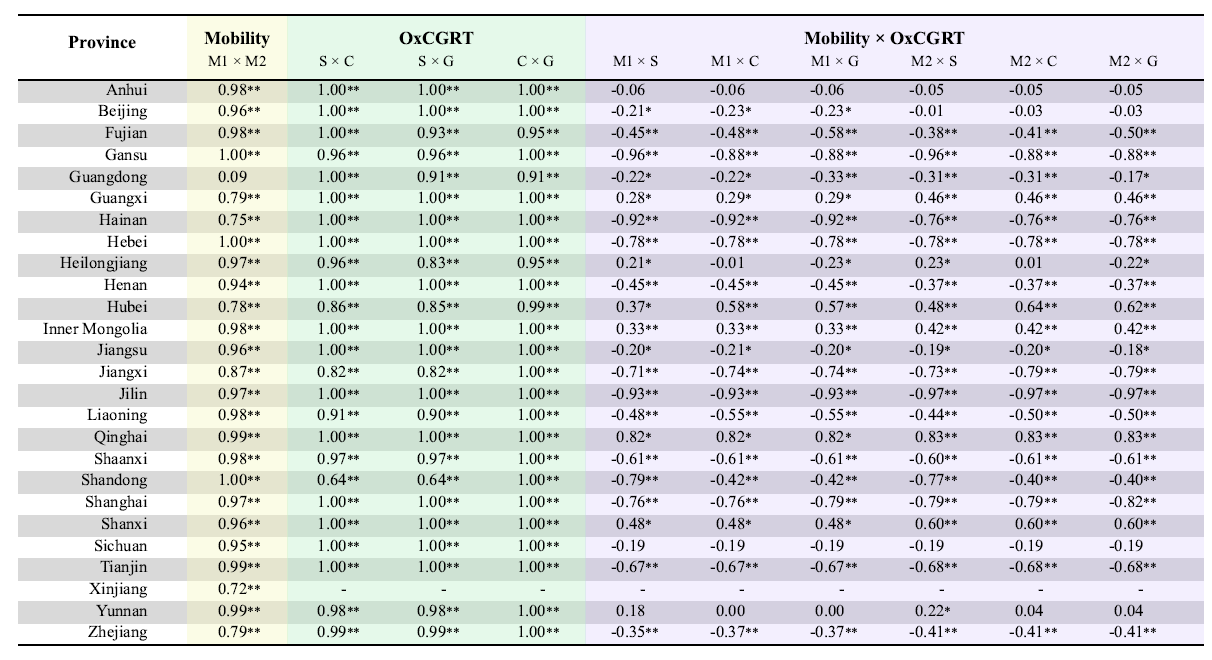


**Table S6**. Comparison of $R_{t}$ estimated based on all cases and$R_{t}^{s}$estimated based on symptomatic cases on province-level. Adjusted *p* value with Benjamini-Hochberg method.


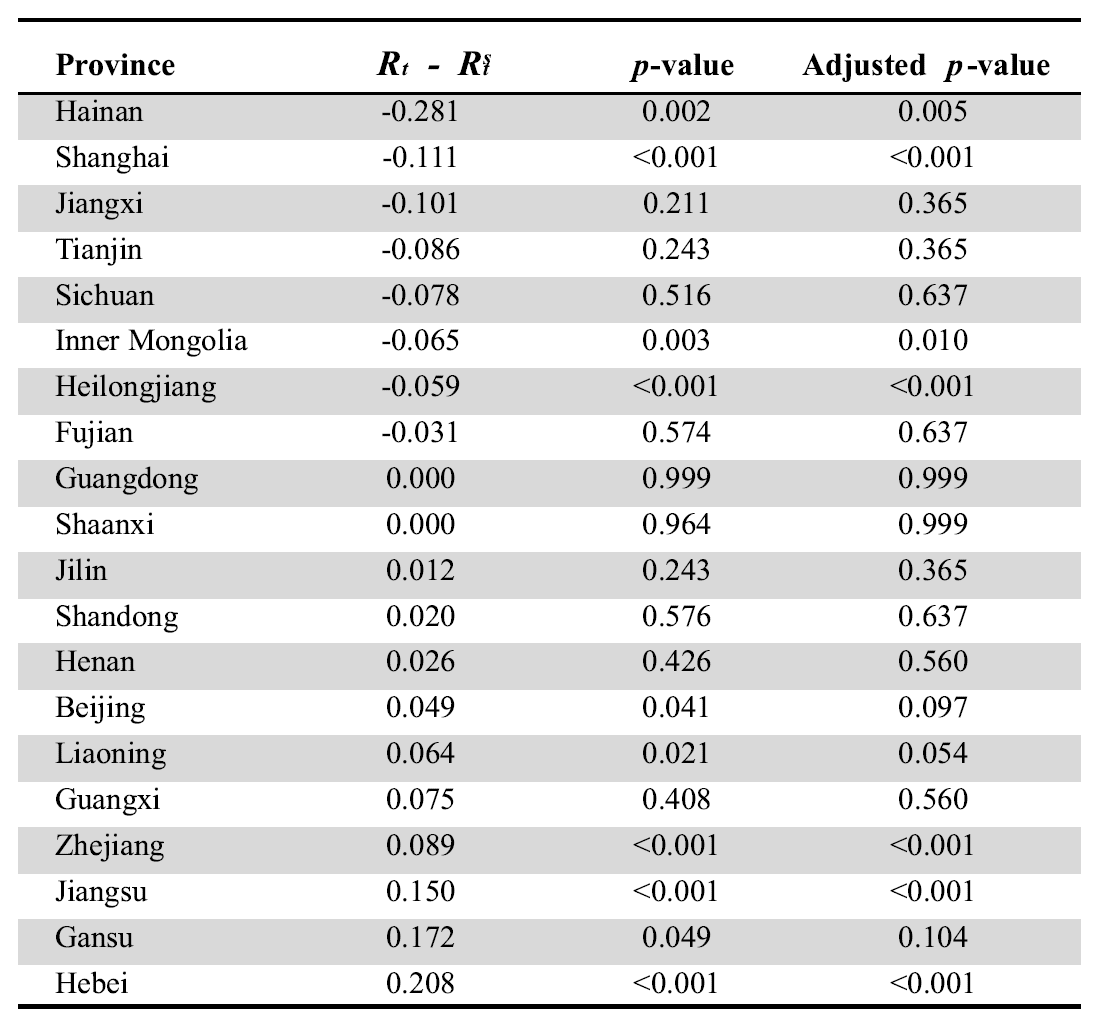


**References**

[1] Cori A, et al. (2013). A new framework and software to estimate time-varying reproduction numbers during epidemics. American Journal of Epidemiology 178:1505-12.

[2] Miller AC, et al. (2022). Statistical Deconvolution for Inference of Infection Time Series. Epidemiology 33:470-9.

[3] Backer JA, et al. (2022). Shorter serial intervals in SARS-CoV-2 cases with Omicron BA.1 variant compared with Delta variant, the Netherlands, 13 to 26 December 2021. Euro Surveillance 27.

[4] The State Council Information Office, The People's Republic of China. China's daily COVID-19 testing capacity hits 15 million. Available at <http://english.scio.gov.cn/pressroom/2021-01/27/content_77160738.htm> (accessed December 2 2022).

[5] The State Council, The People's Republic of China. China nucleic acid testing capacity at 51.65m samples a day. Available at <https://english.www.gov.cn/news/topnews/202204/18/content_WS625cb6d7c6d02e5335329763.html> (accessed December 2 2022).

[6] Department of Science and Technology of Guangdong Province. <http://gdstc.gd.gov.cn/kjfyzl/gnwdt/content/post_3261369.html>. (accessed November 30 2022).

[7] The New York Times. In Its War on Covid-19, China Calls on ‘Little Inoculated Warriors’. Available at <https://www.nytimes.com/2021/12/06/business/china-covid-vaccine-children.html?_ga=2.1079478.605070662.1669704296-1034177539.1669704296> (accessed November 30 2022).

[8] National Health Commission of the People’s Republic of China. Joint Prevention and Control Mechanism Press Conference on 13 August 2021. Available at <http://www.nhc.gov.cn/xcs/fkdt/202108/6c77e46253b84b1f9cca2d5aa4a0f76b.shtml> (accessed November 29 2022).

[9] National Health Commission of the People's Republic of China. Joint Prevention and Control Mechanism Press Conference on 28 April 2022. Available at <http://www.nhc.gov.cn/xcs/fkdt/202204/34ce2f4566844cee9c0b2f0a5afbf6f7.shtml> (accessed December 2 2022).

[10] Media C. Available at <https://china.caixin.com/2022-11-29/101971919.html> (accessed December 3 2022).

[11] VIPER Group COVID19 Vaccine Tracker Team. COVID19 Vaccine Tracker. Available at <https://covid19.trackvaccines.org/country/china/> (accessed December 7 2023).
