# Supplementary figures and images for: Comparative epidemiology of outbreaks caused by SARS-CoV-2 Delta and Omicron variants in China
Source: Epidemiol Infect. 2024 Mar 19;152:e43. doi: 10.1017/S0950268824000360 (PMC10964185; doi:10.1017/S0950268824000360)

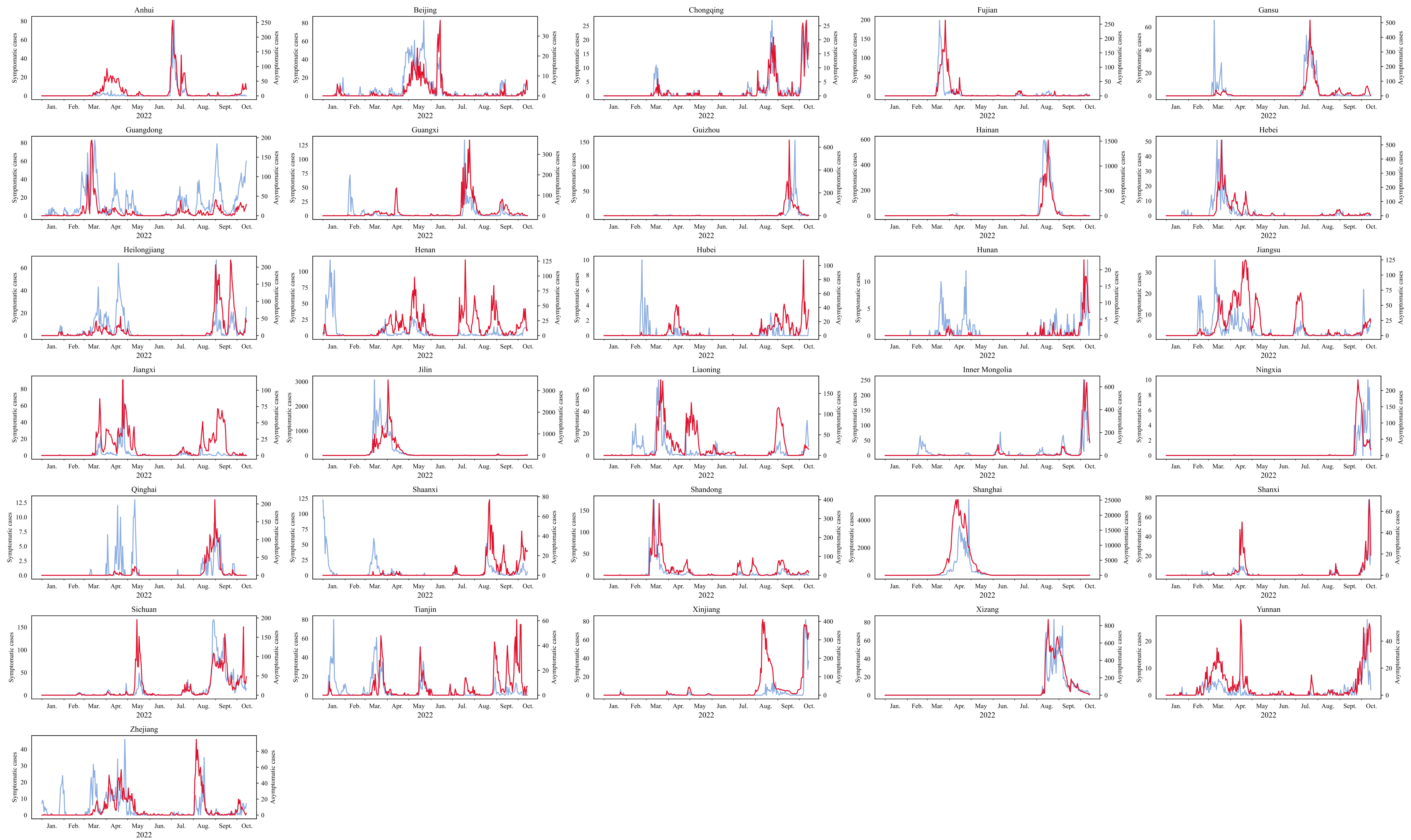

Supplement: Peng et al. supplementary material 2 — Peng et al. supplementary material [file S0950268824000360sup002.pdf]

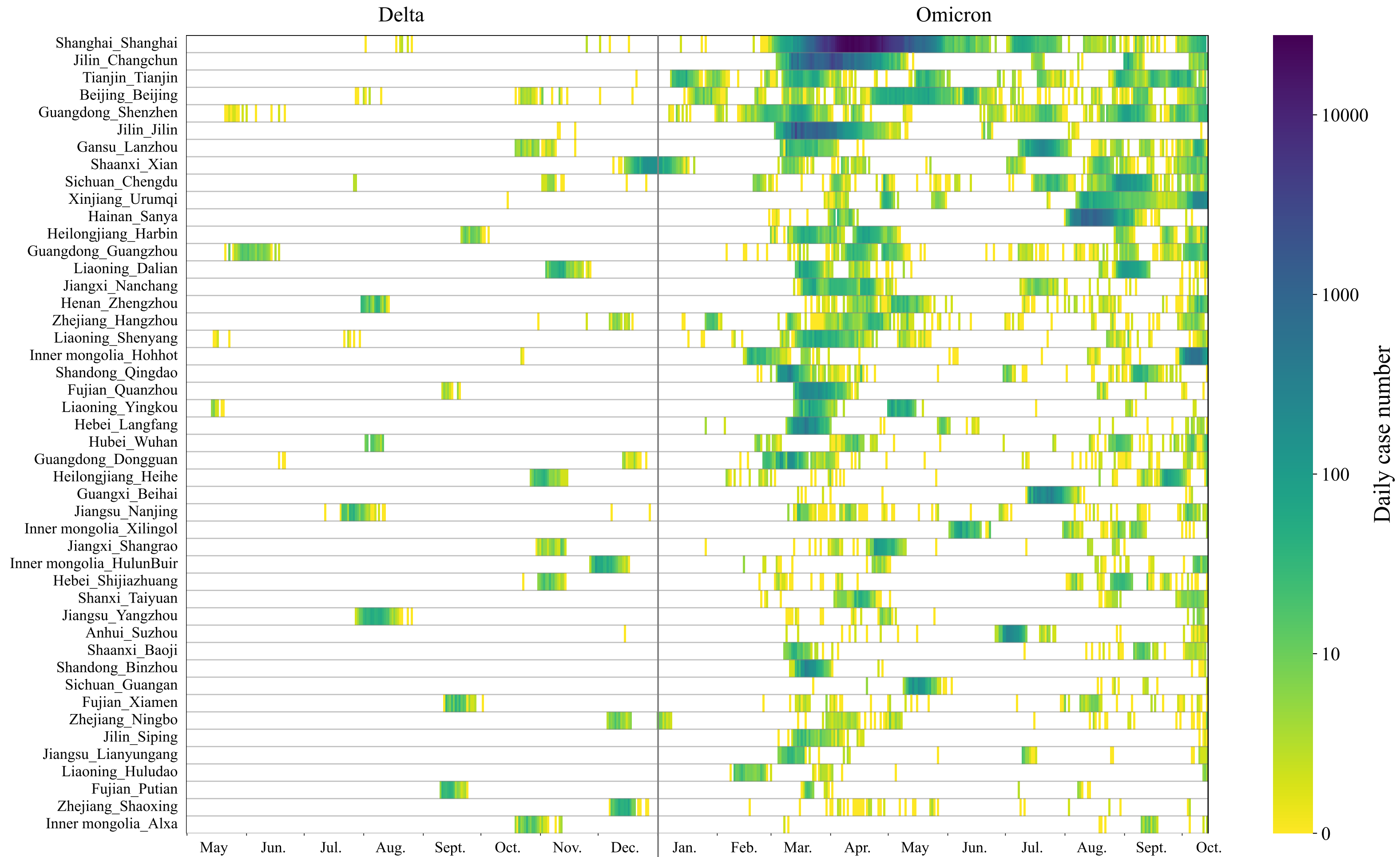

Supplement: Peng et al. supplementary material 3 — Peng et al. supplementary material [file S0950268824000360sup003.pdf]

### A. Inter-city inflow

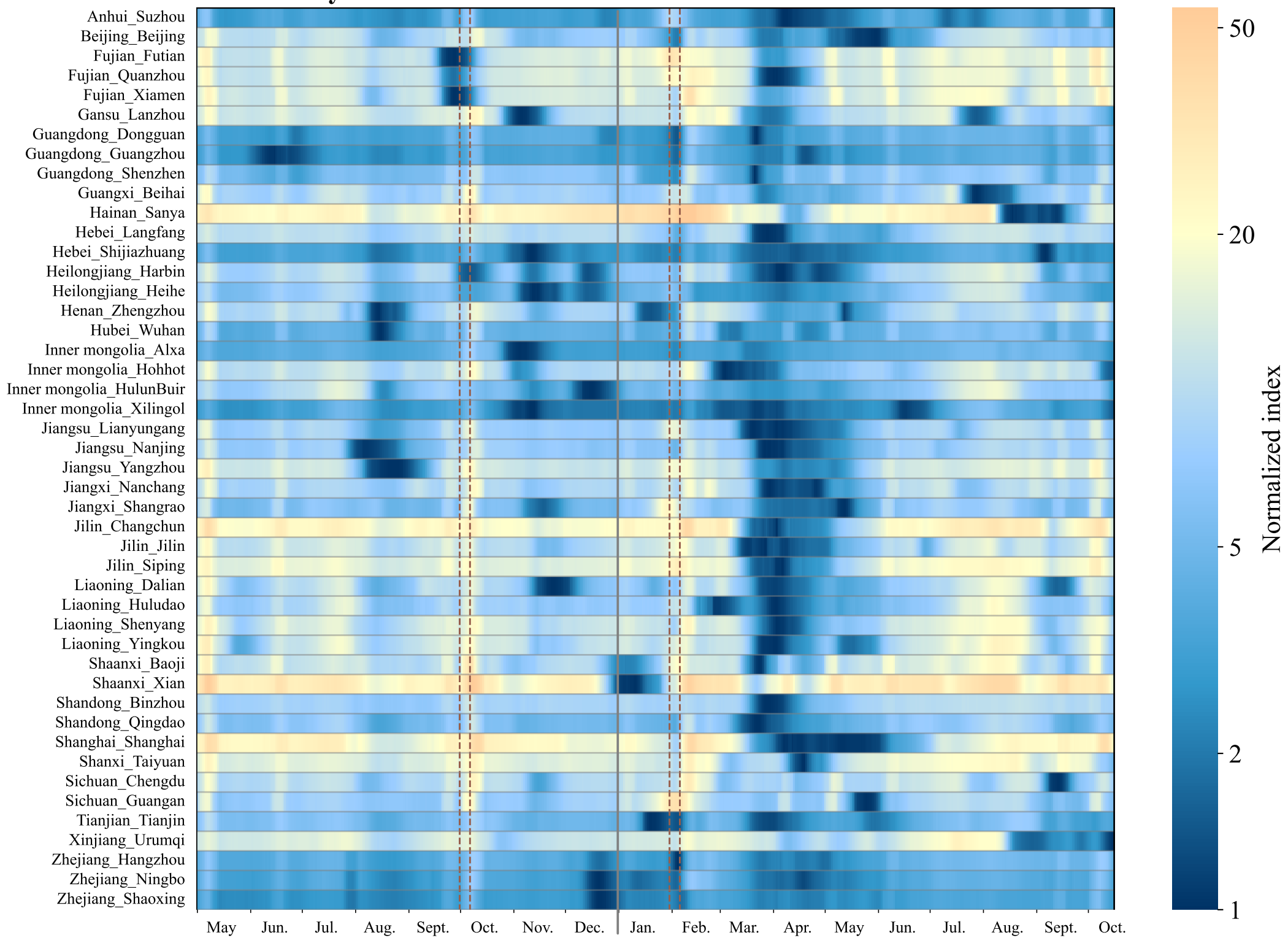

### B. Inter-city outflow

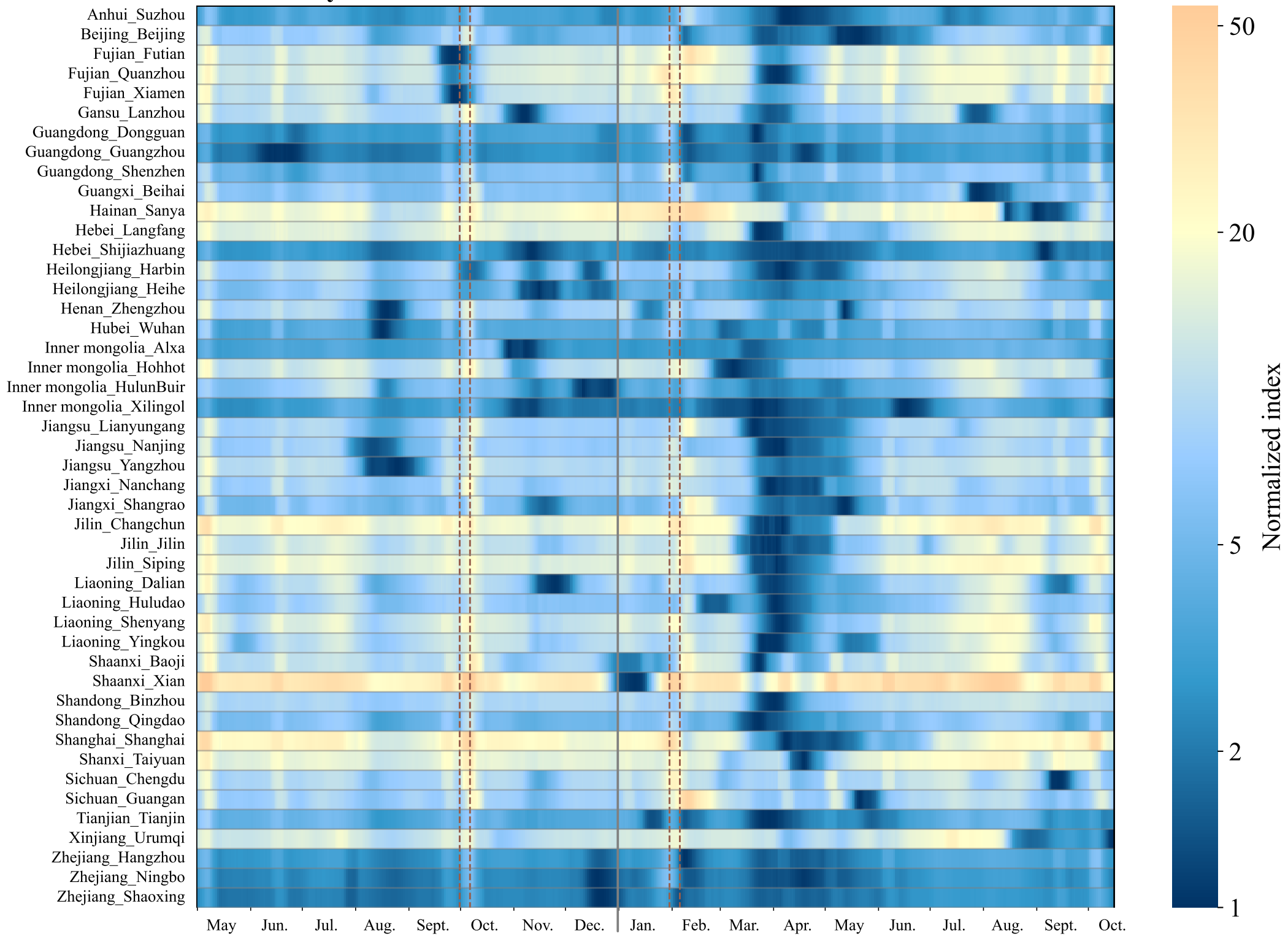

Supplement: Peng et al. supplementary material 4 — Peng et al. supplementary material [file S0950268824000360sup004.pdf]

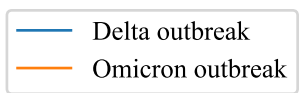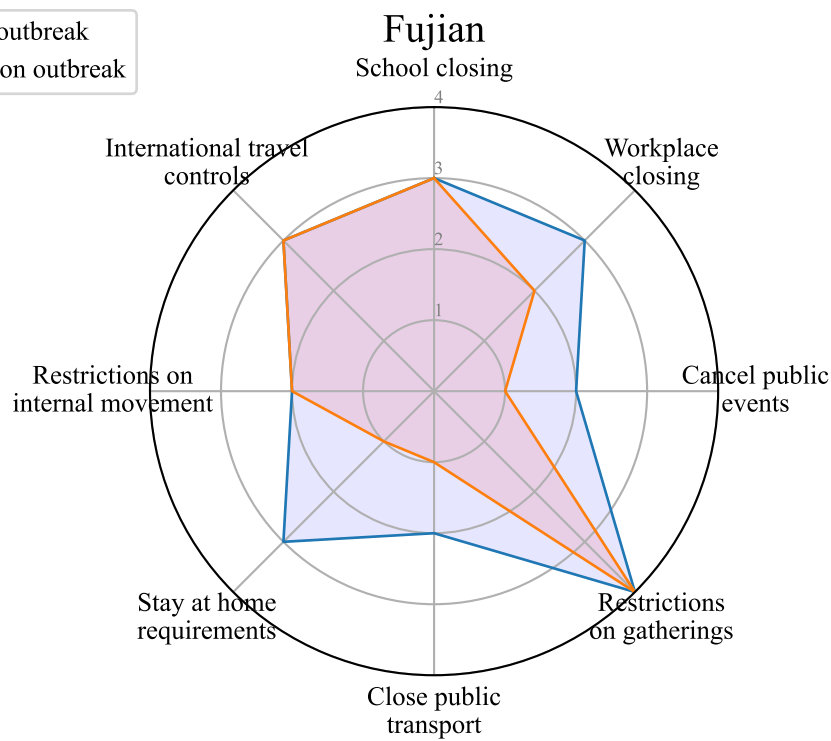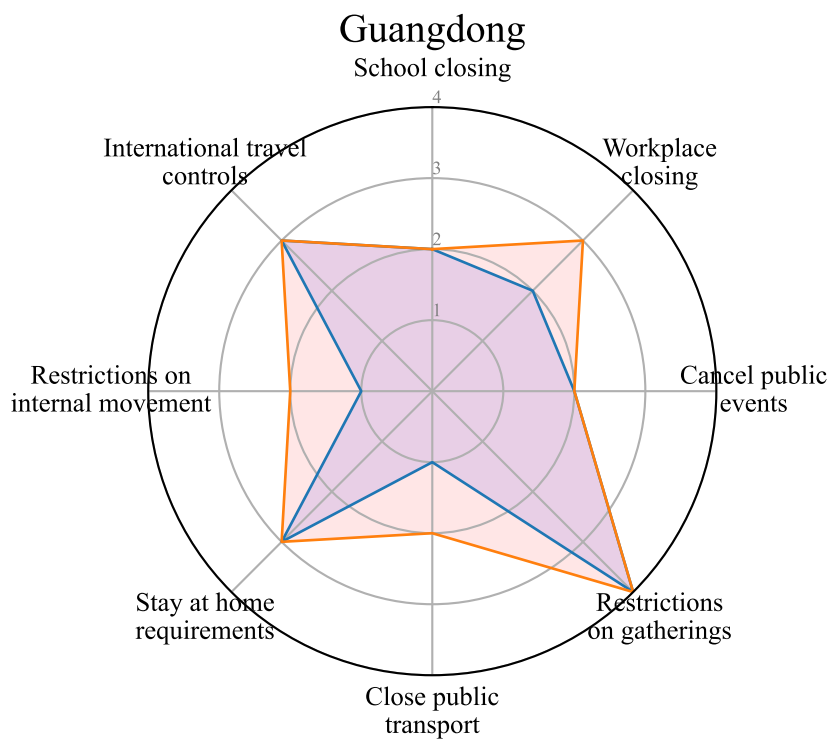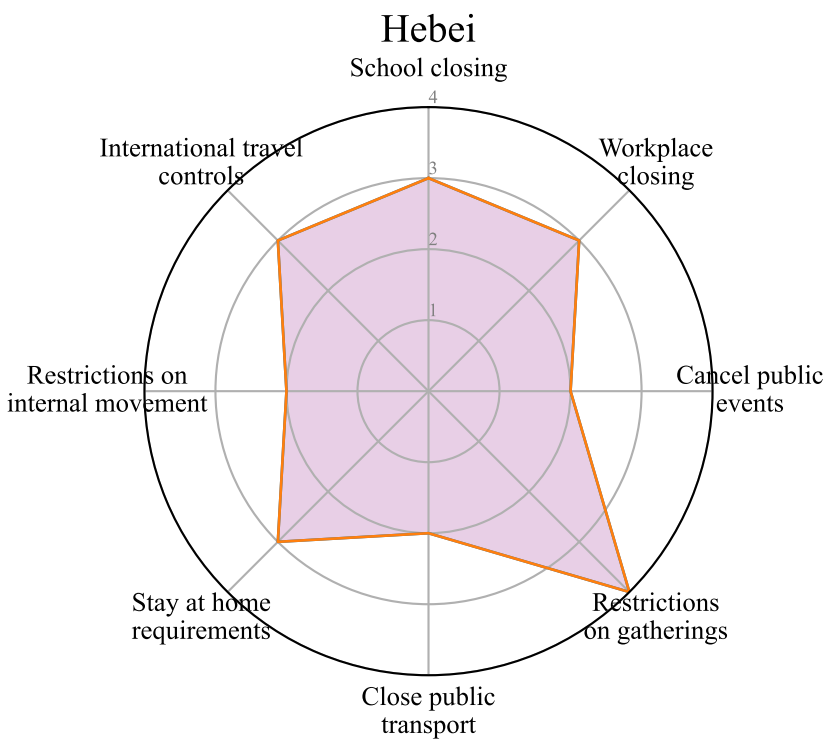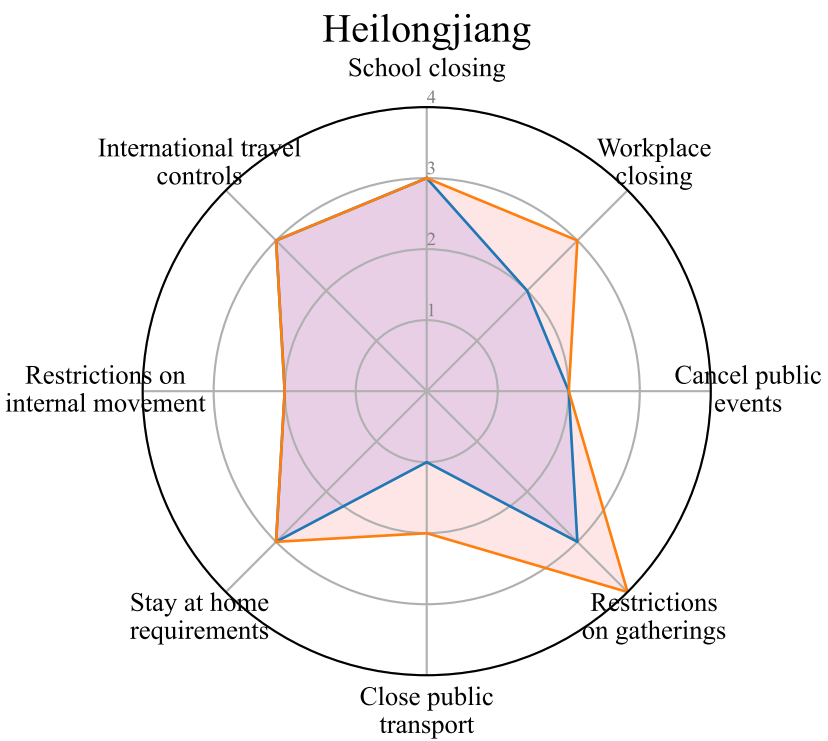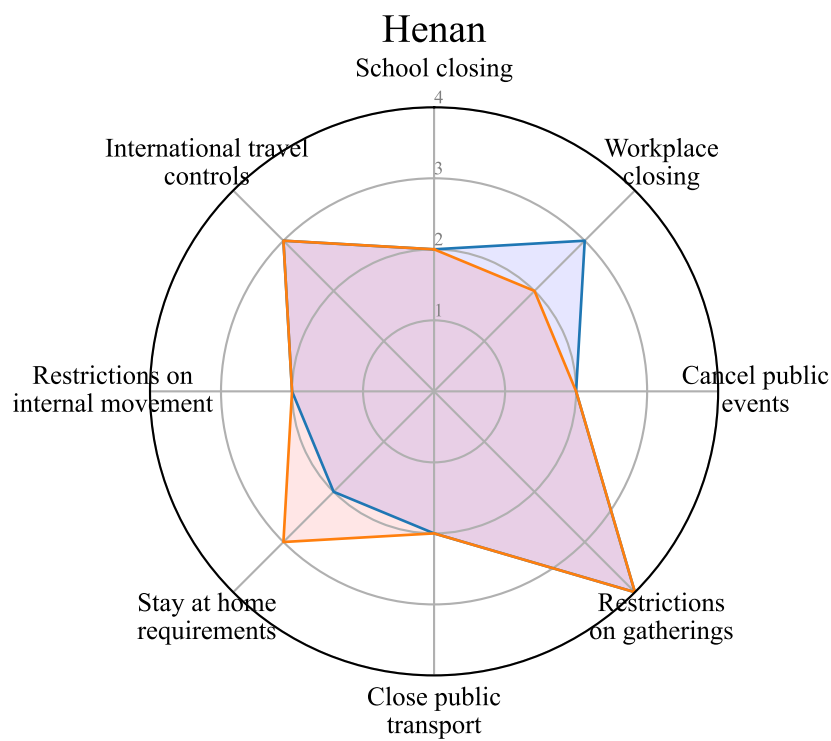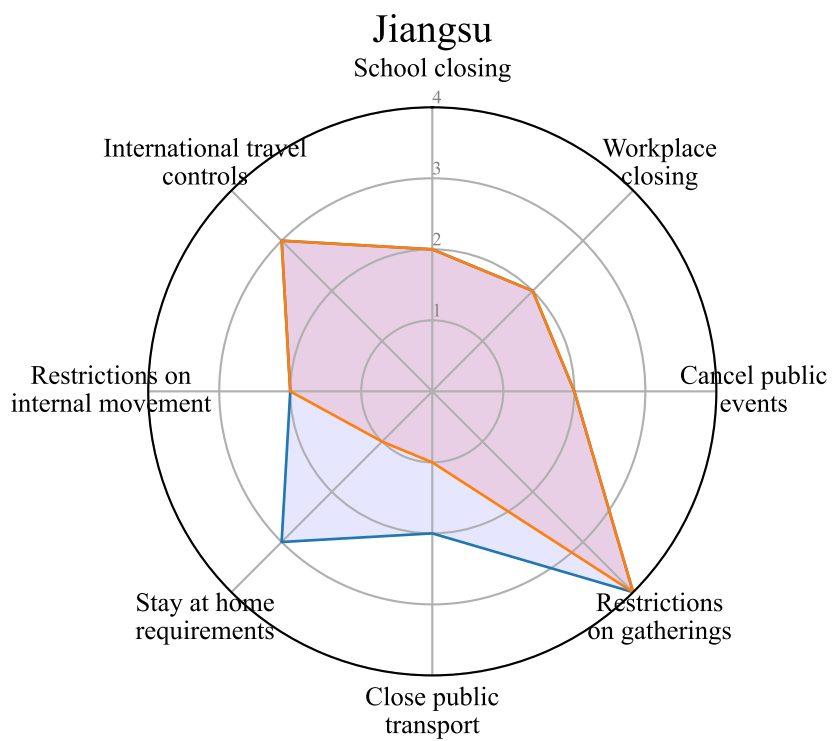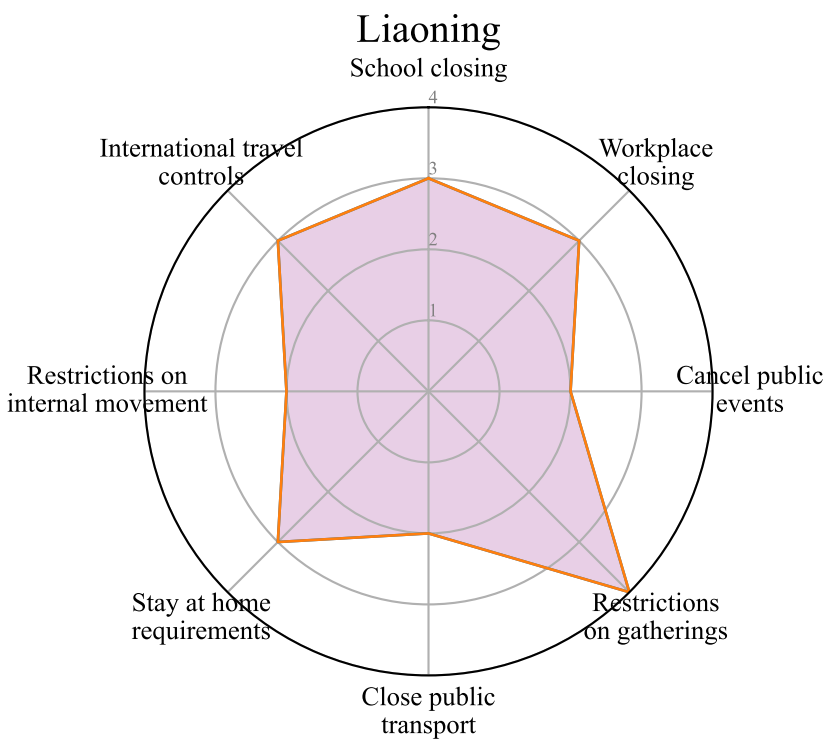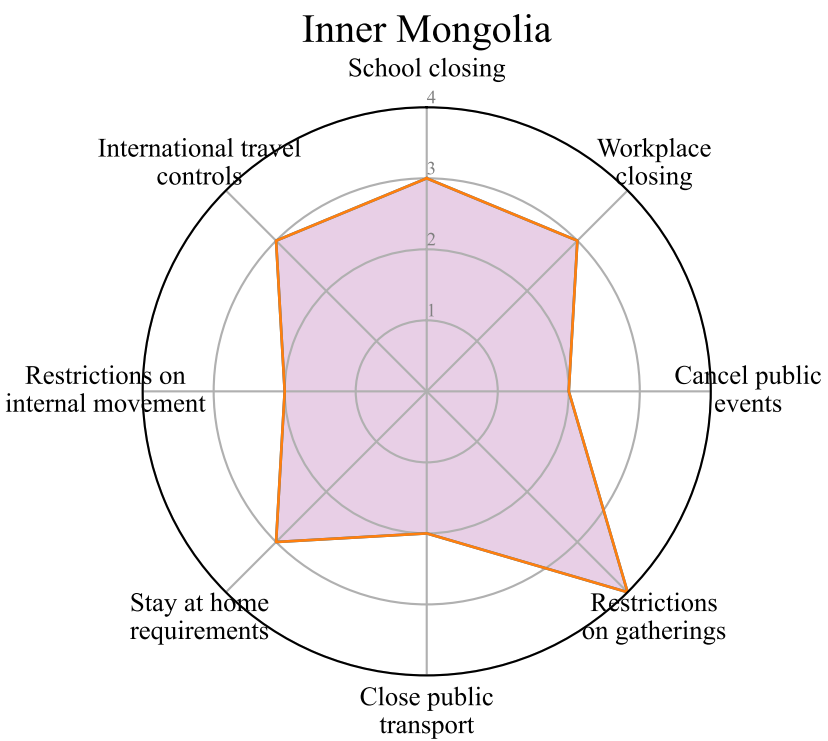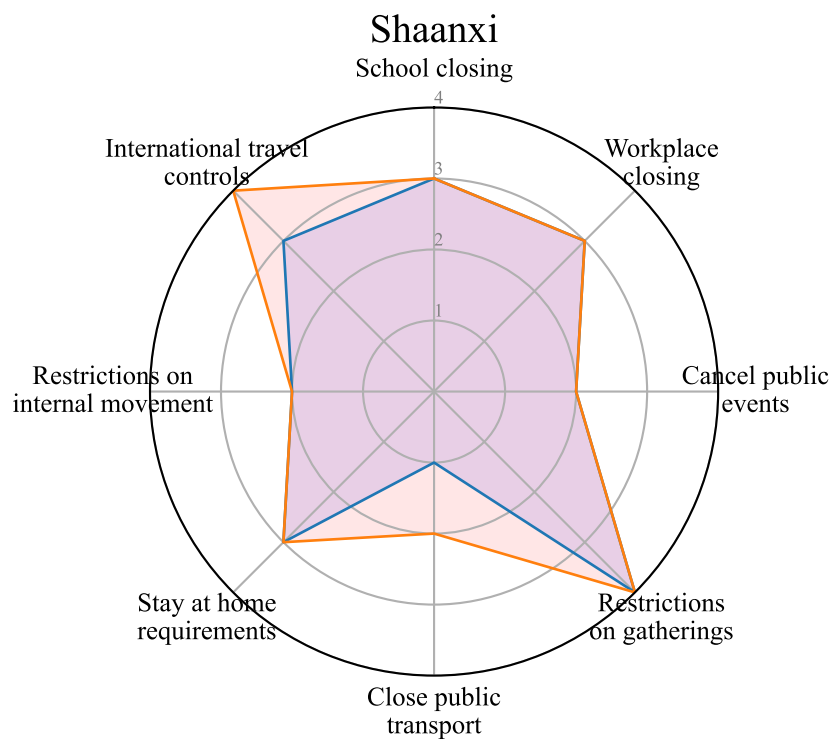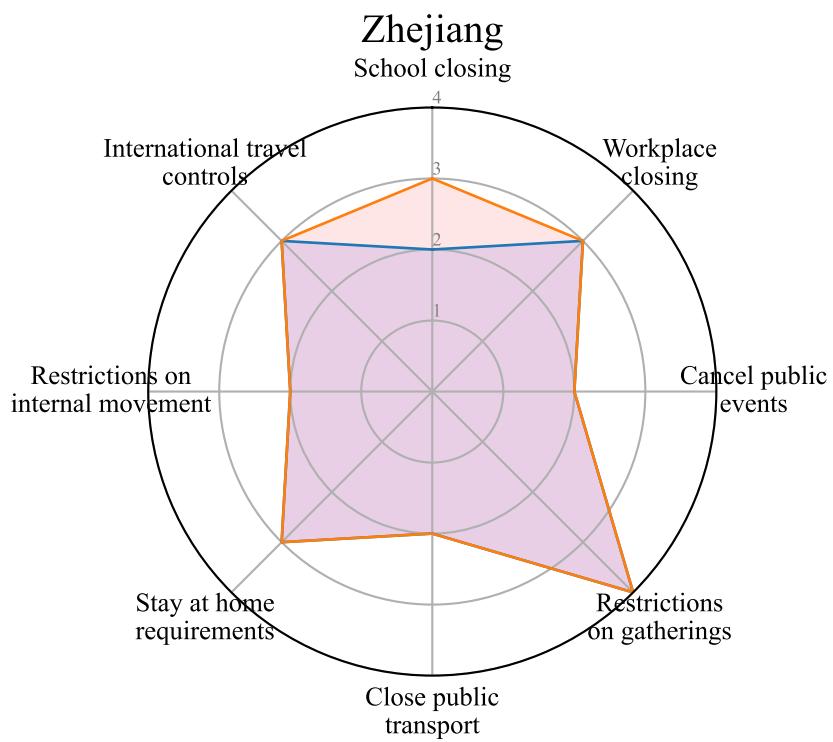

Supplement: Peng et al. supplementary material 5 — Peng et al. supplementary material [file S0950268824000360sup005.pdf]

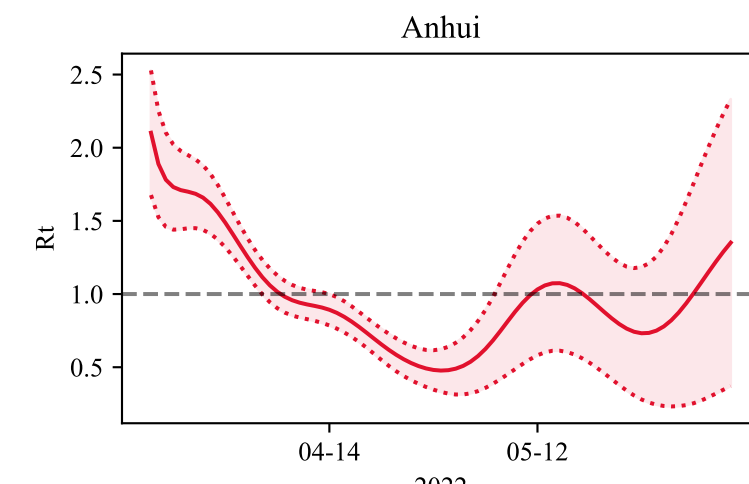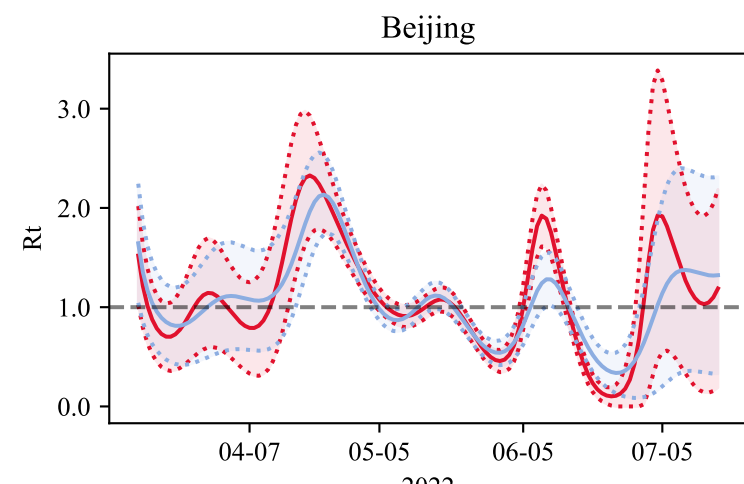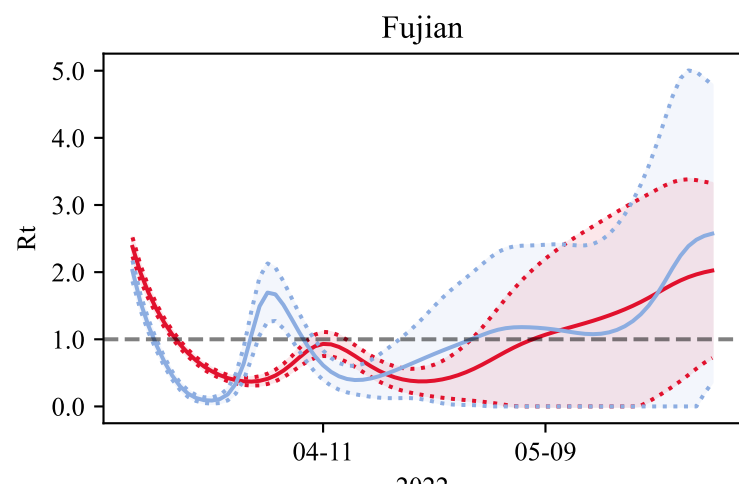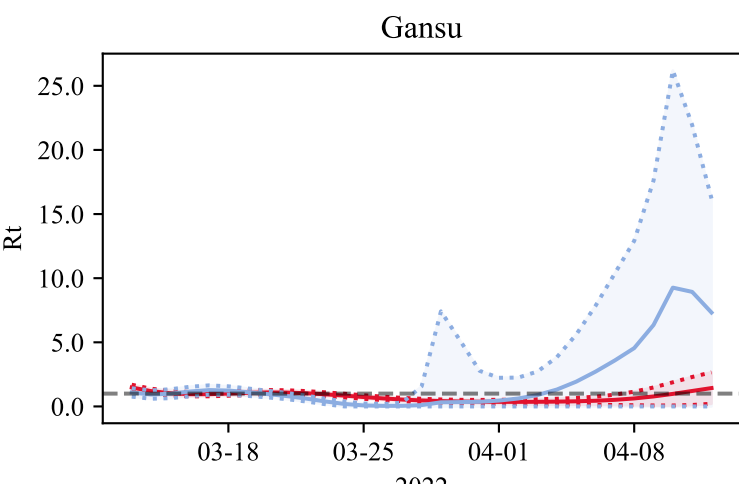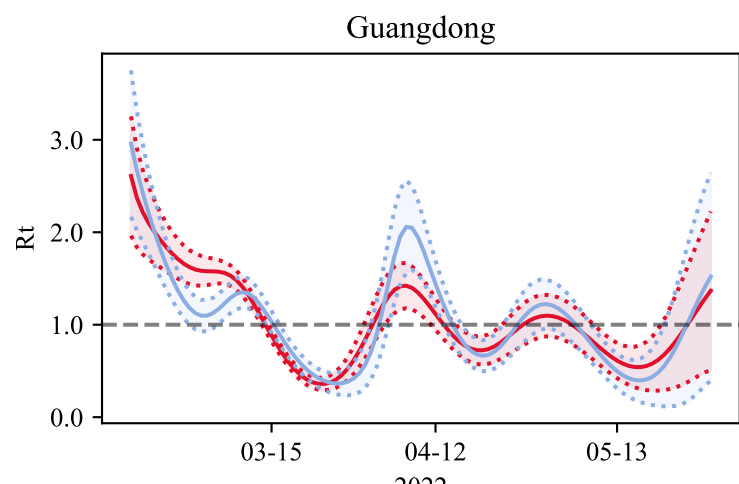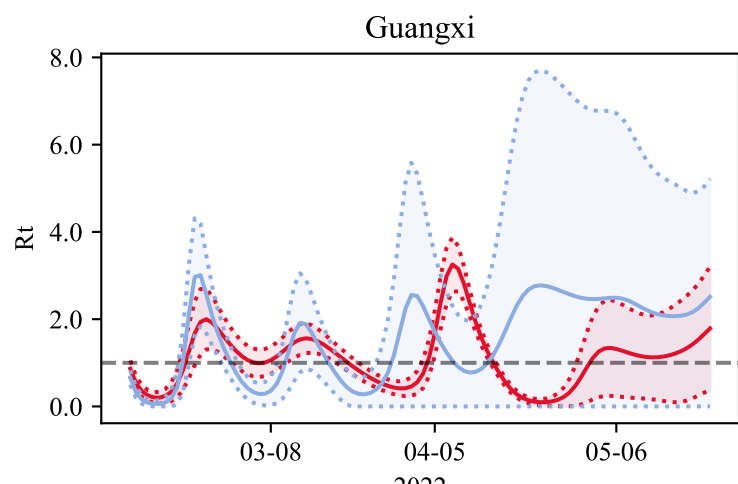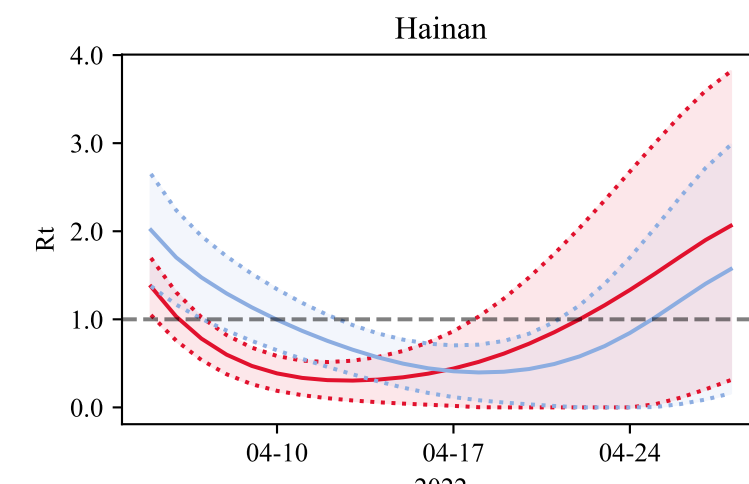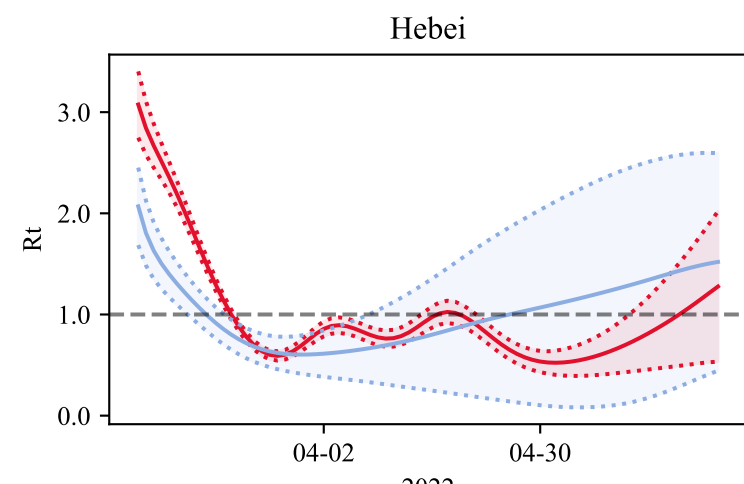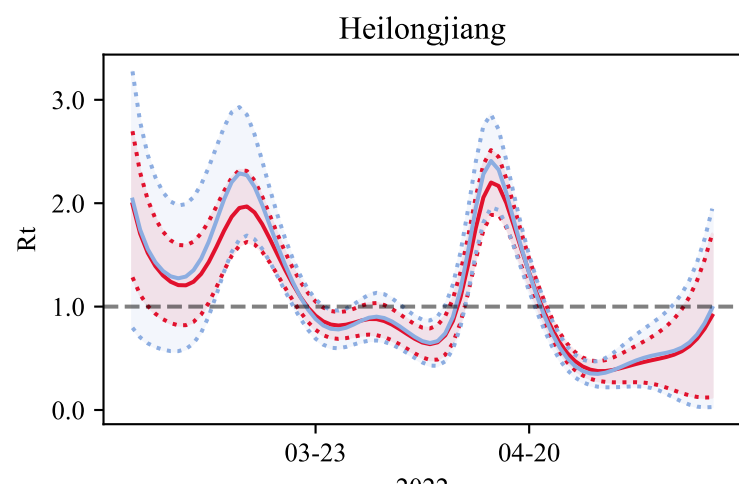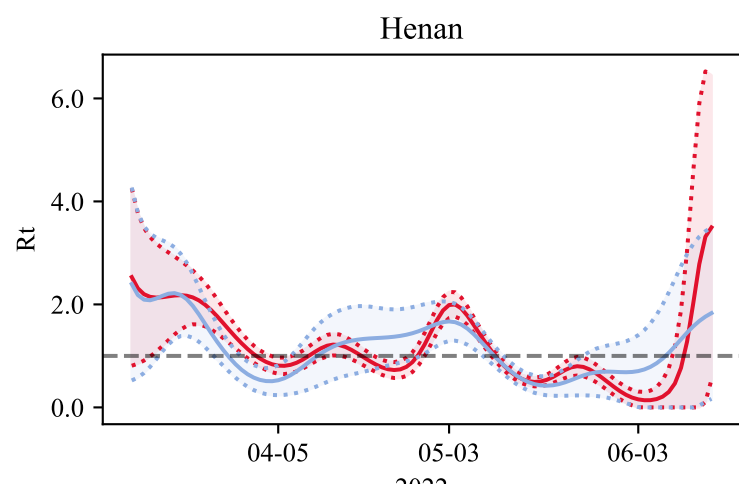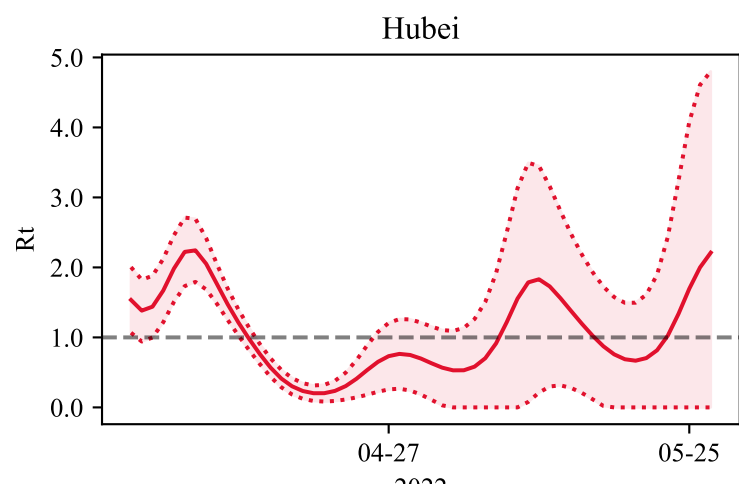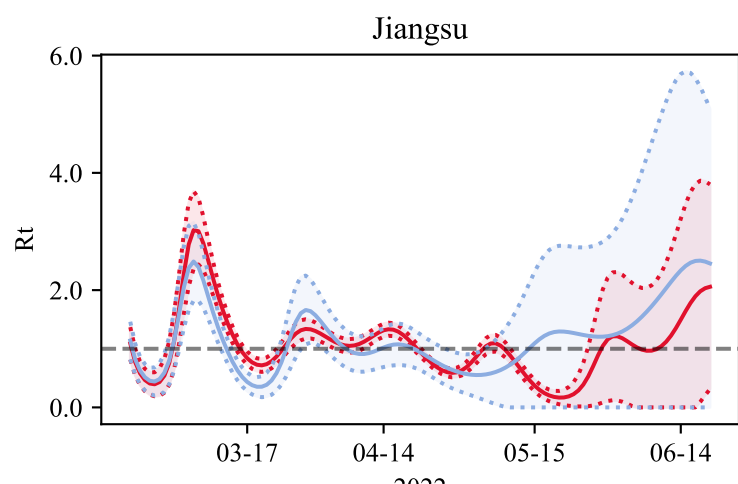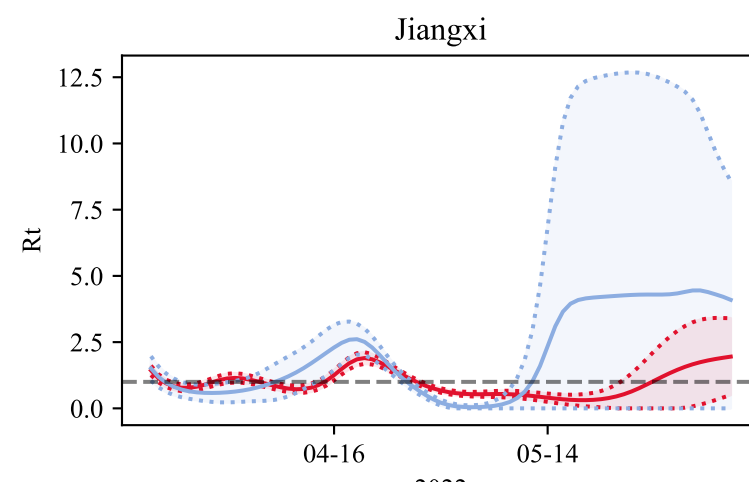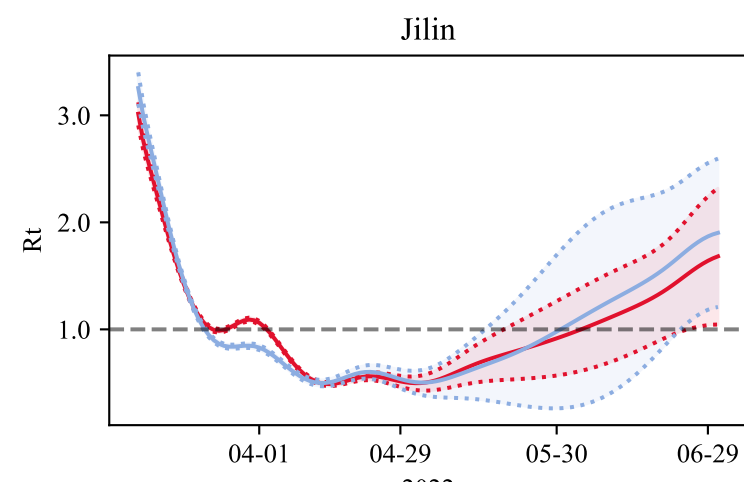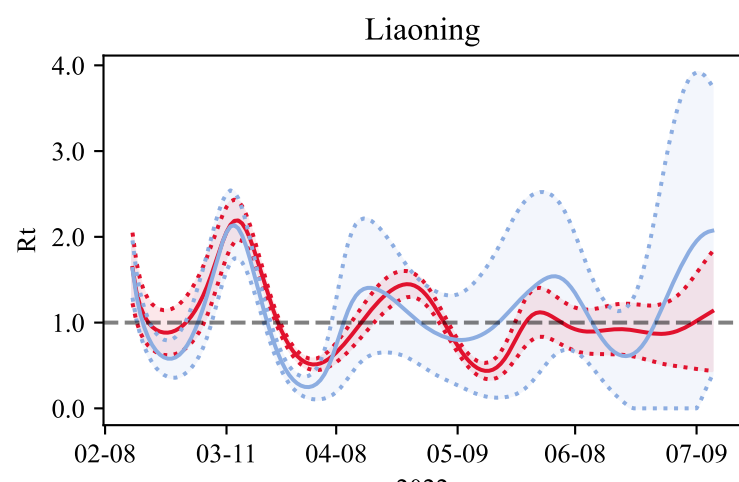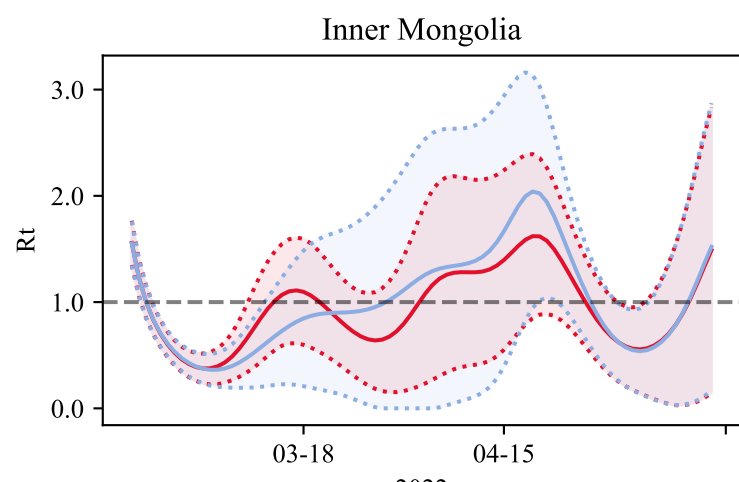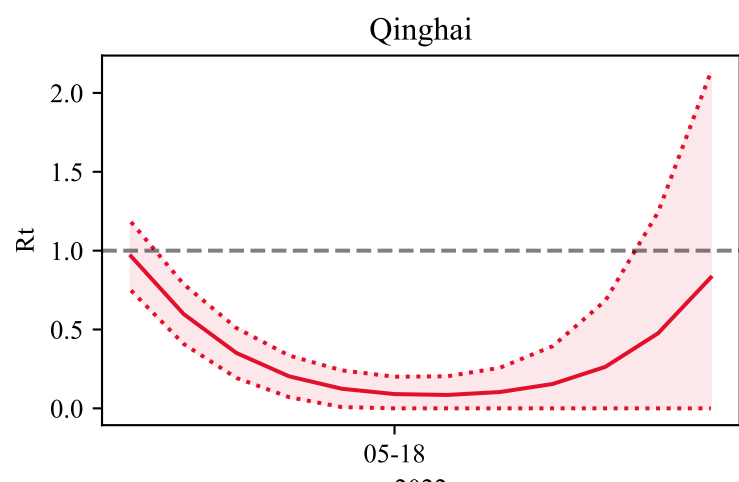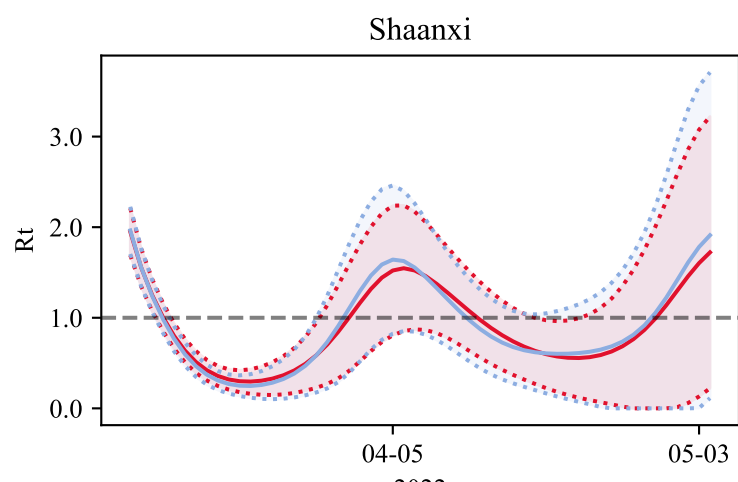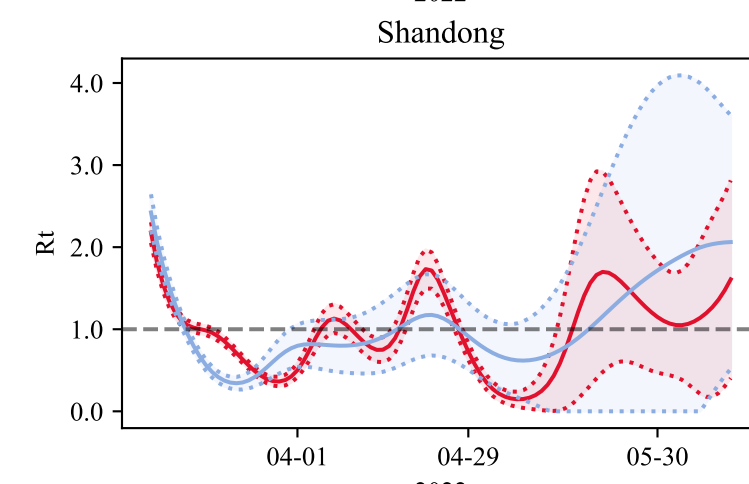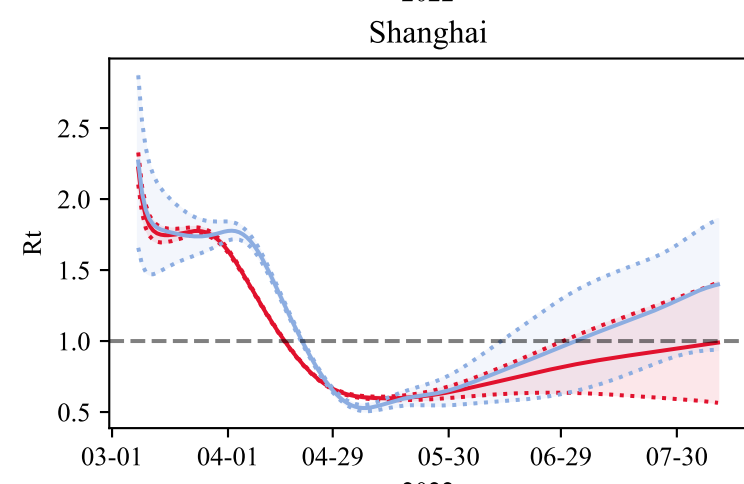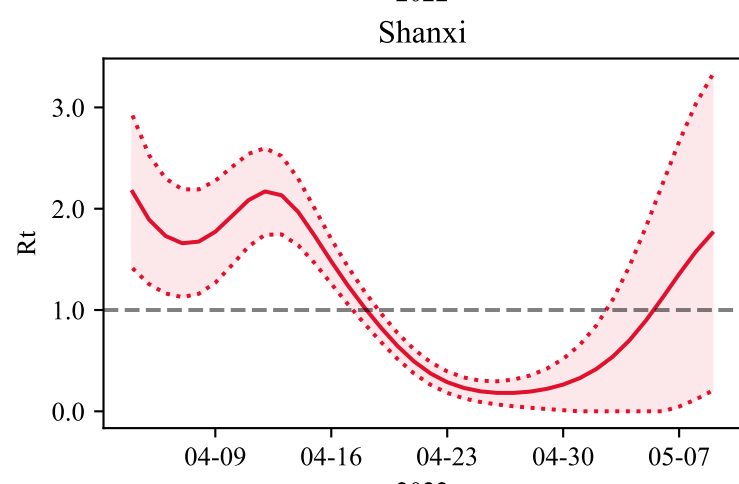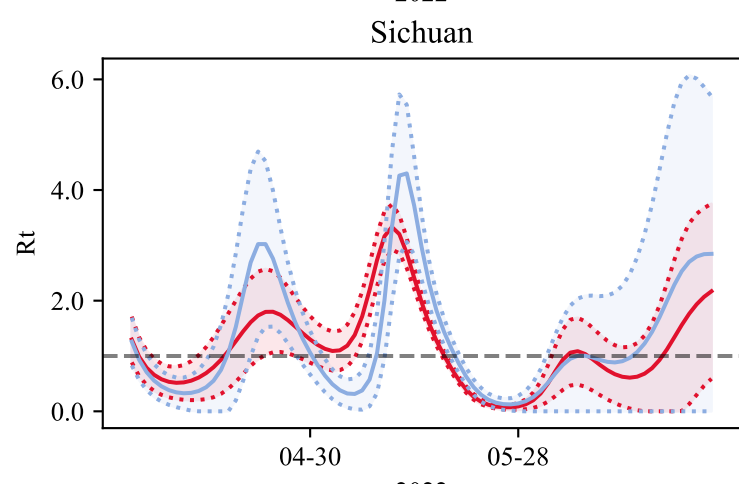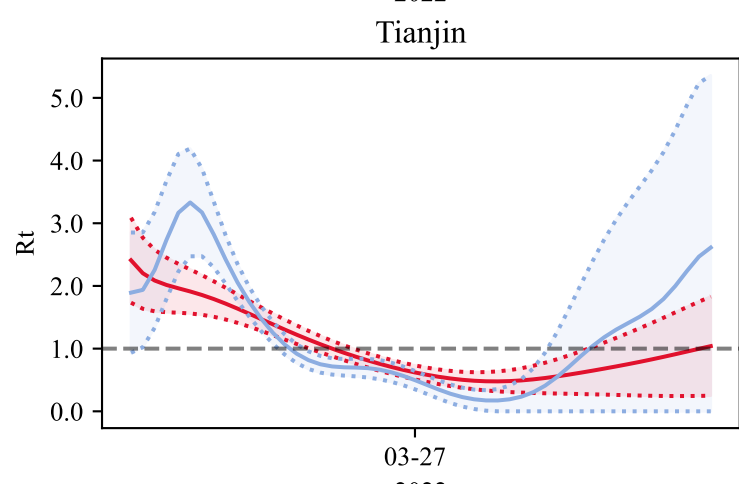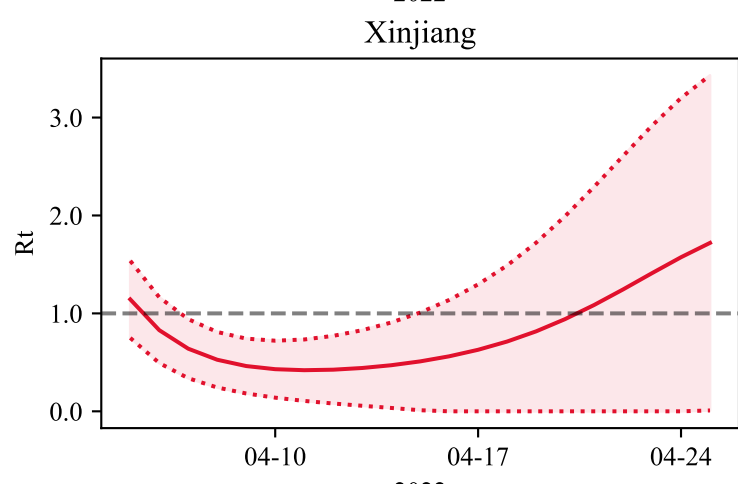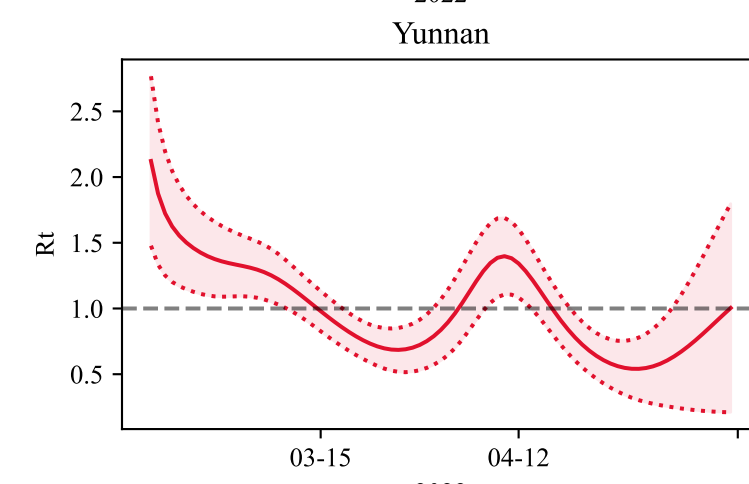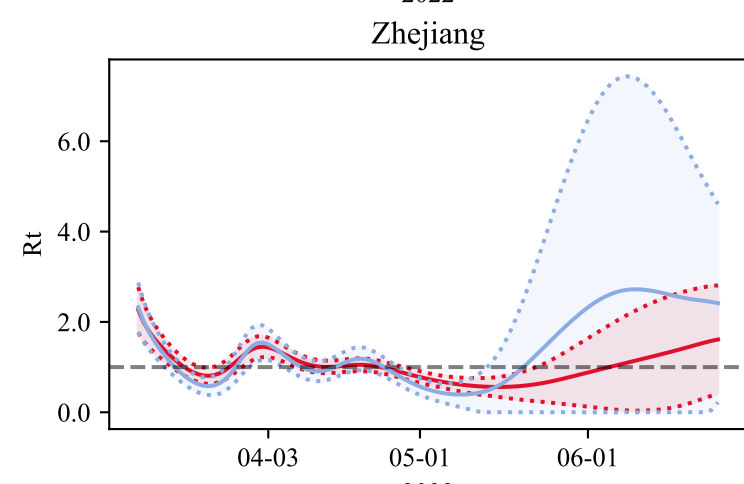

Supplement: Peng et al. supplementary material 6 — Peng et al. supplementary material [file S0950268824000360sup006.pdf]

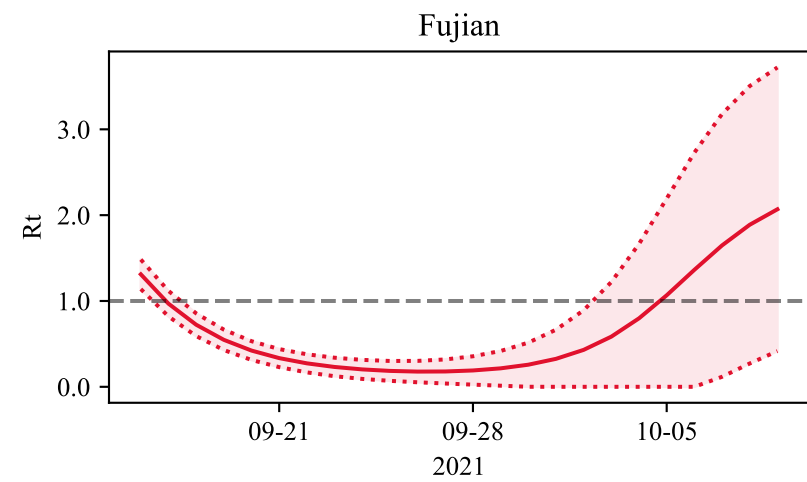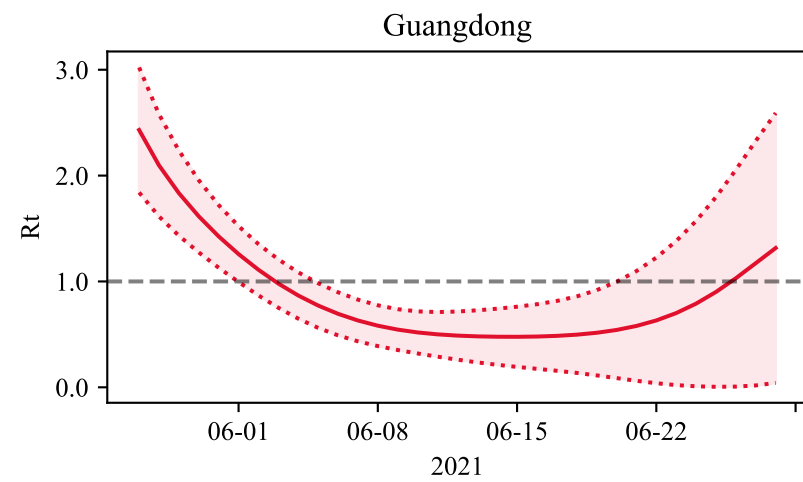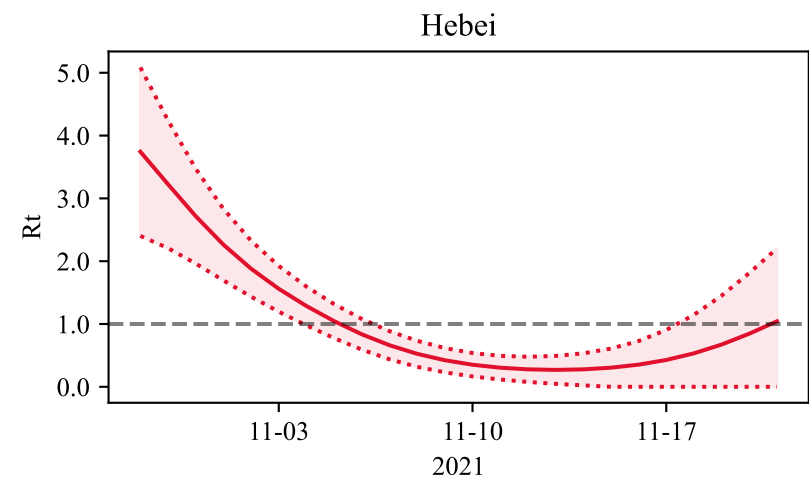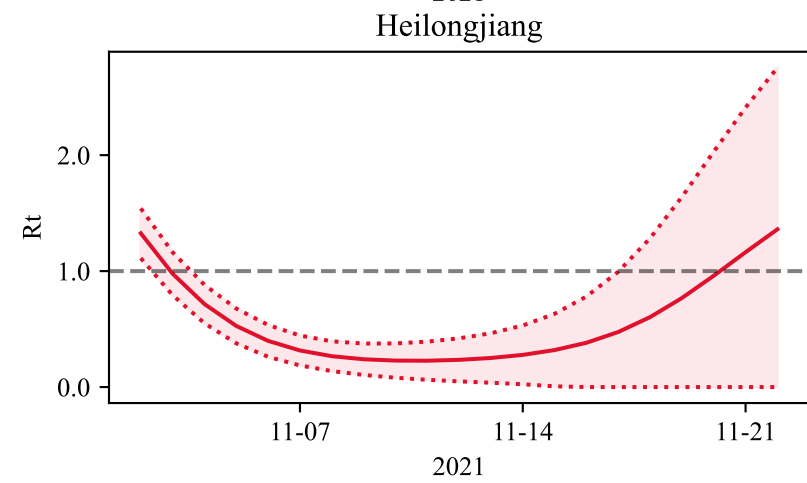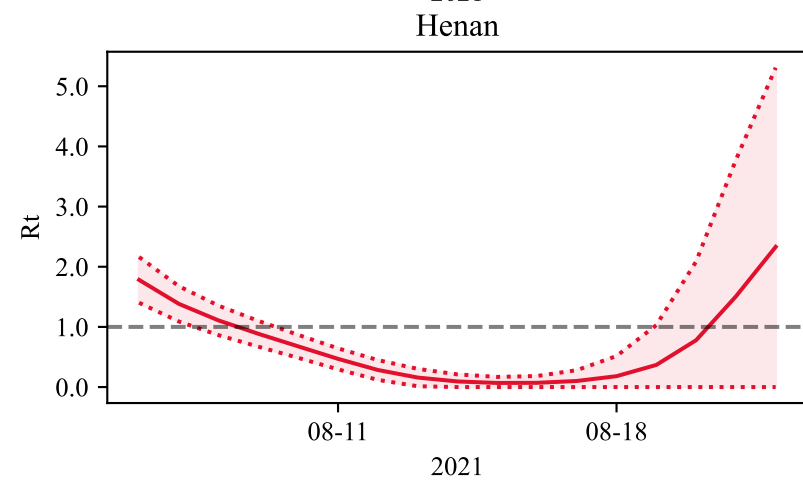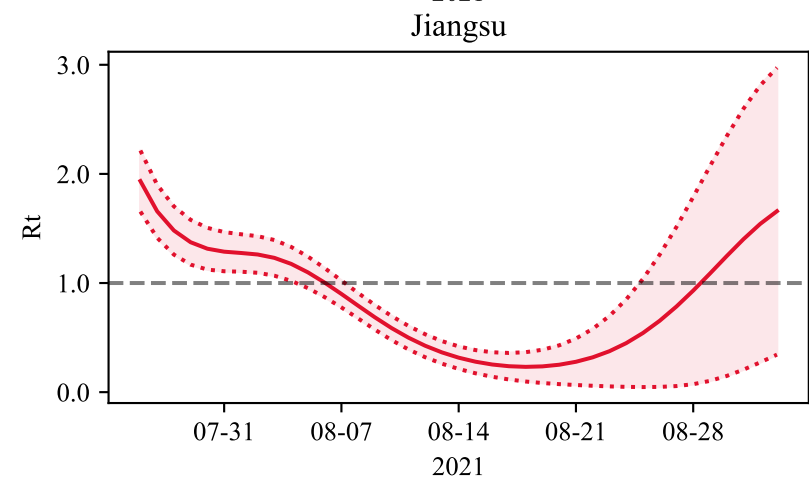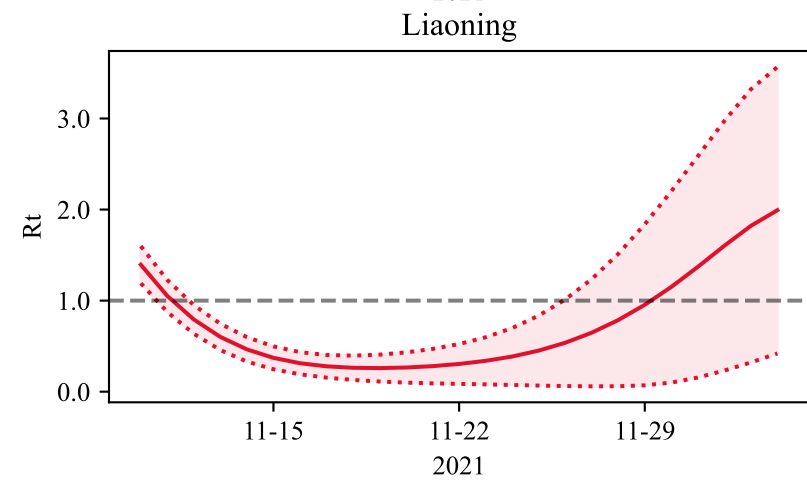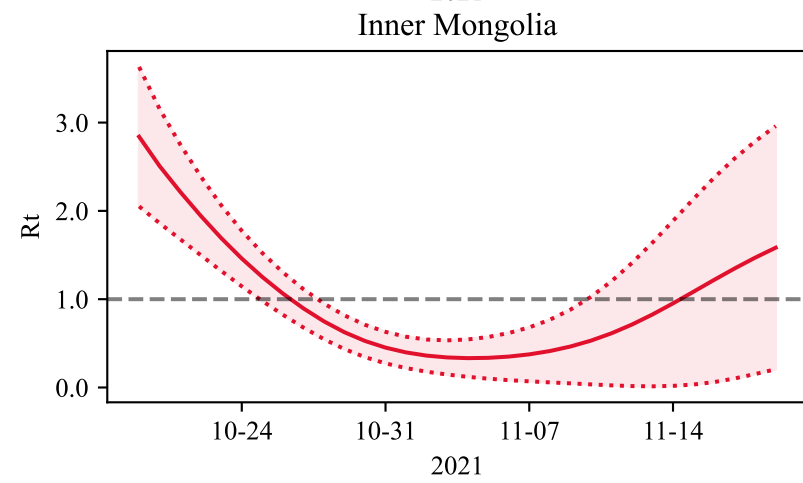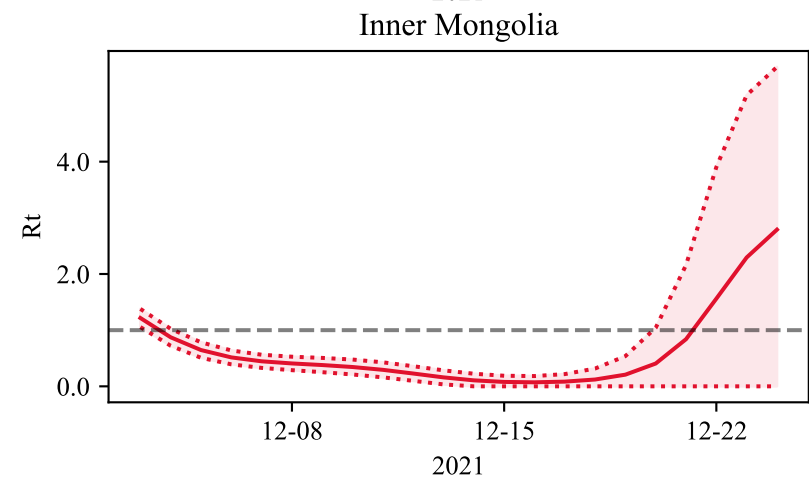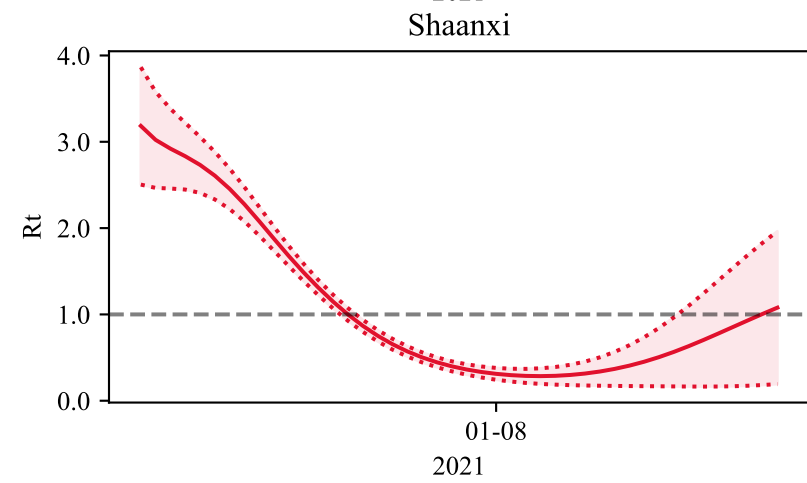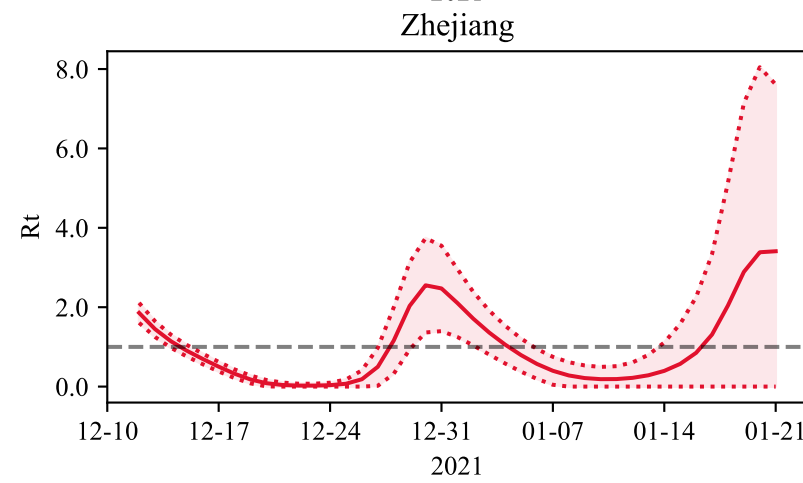

Supplement: Peng et al. supplementary material 7 — Peng et al. supplementary material [file S0950268824000360sup007.pdf]

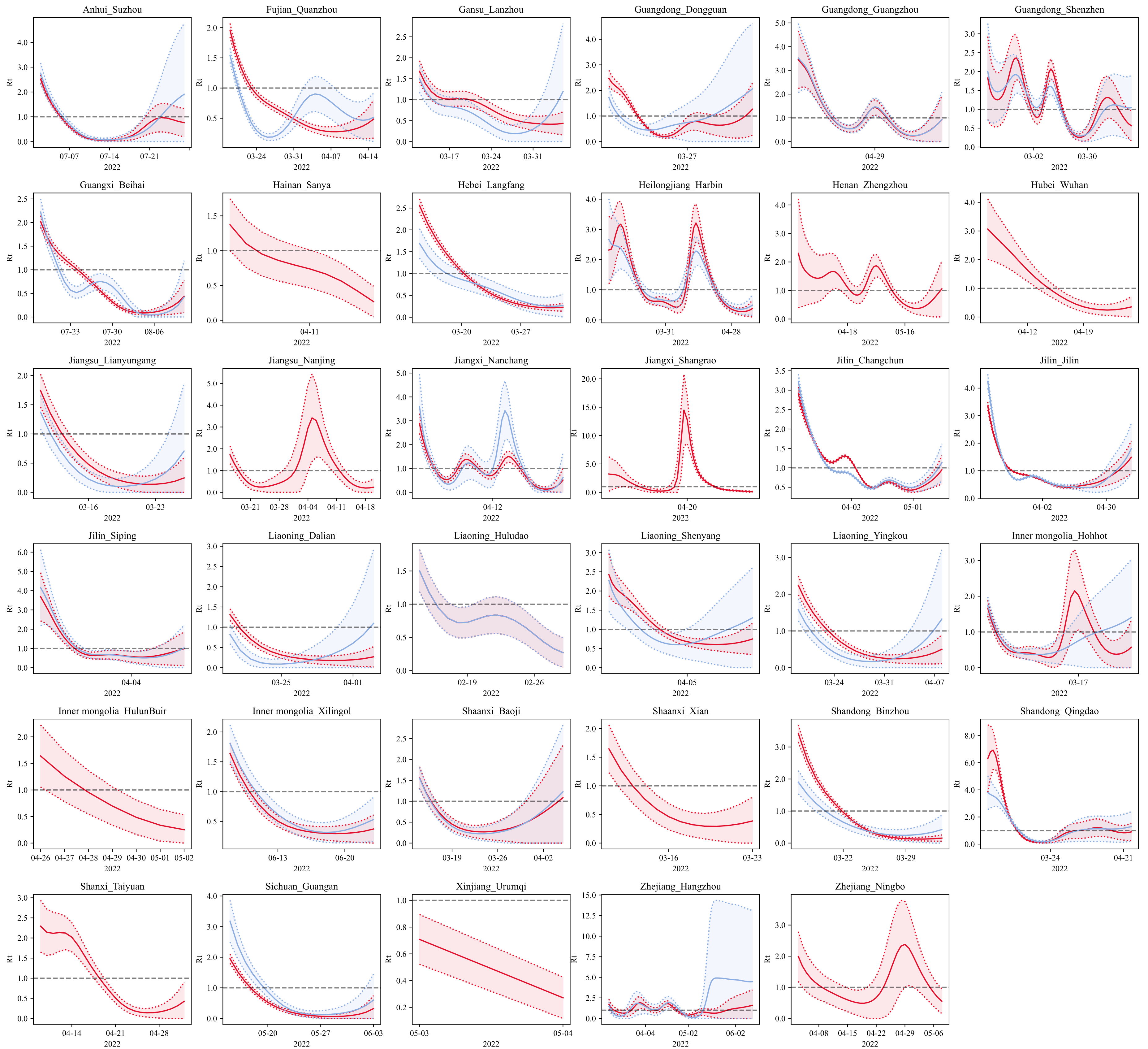

Supplement: Peng et al. supplementary material 8 — Peng et al. supplementary material [file S0950268824000360sup008.pdf]

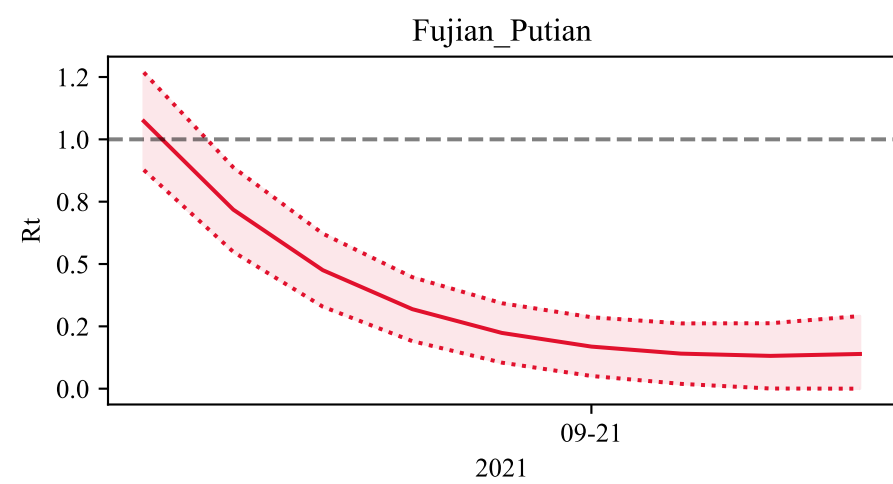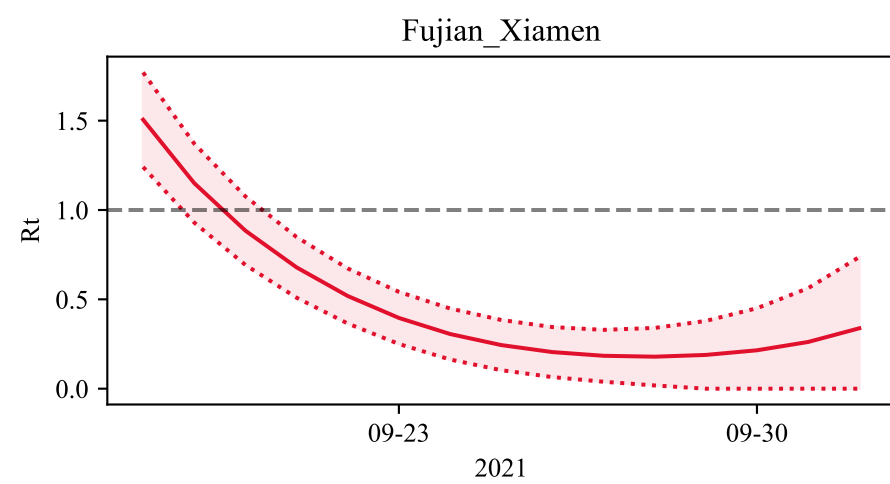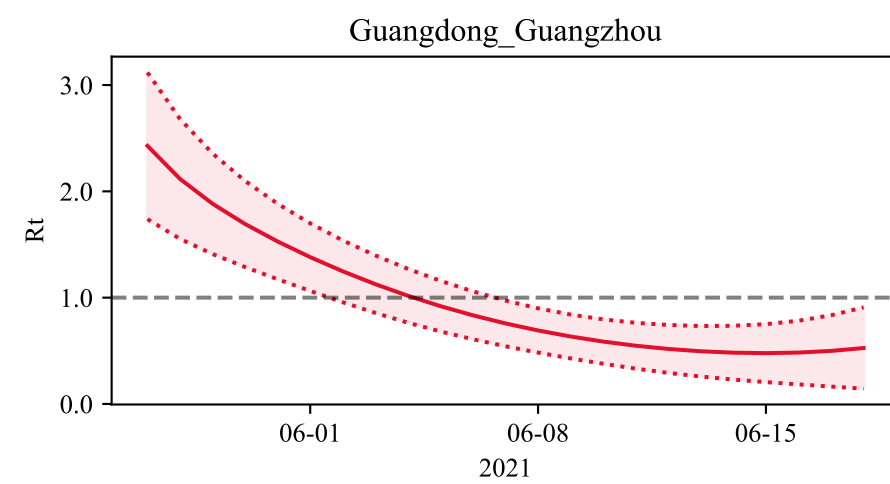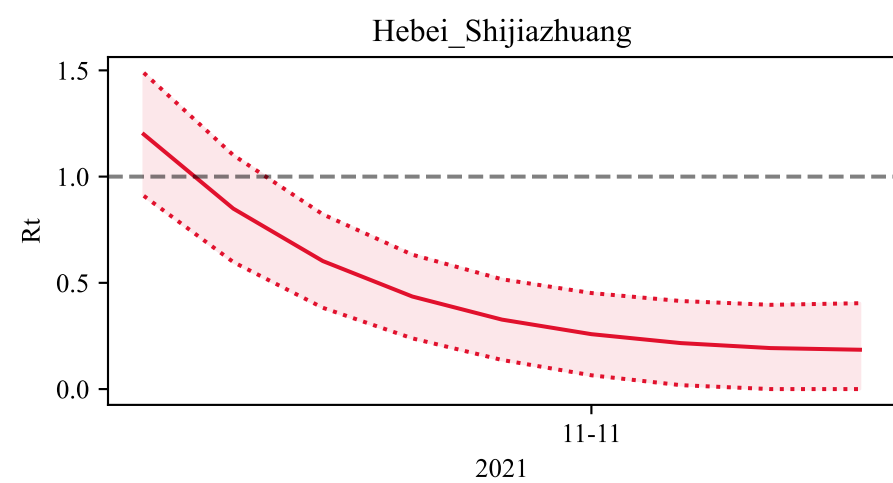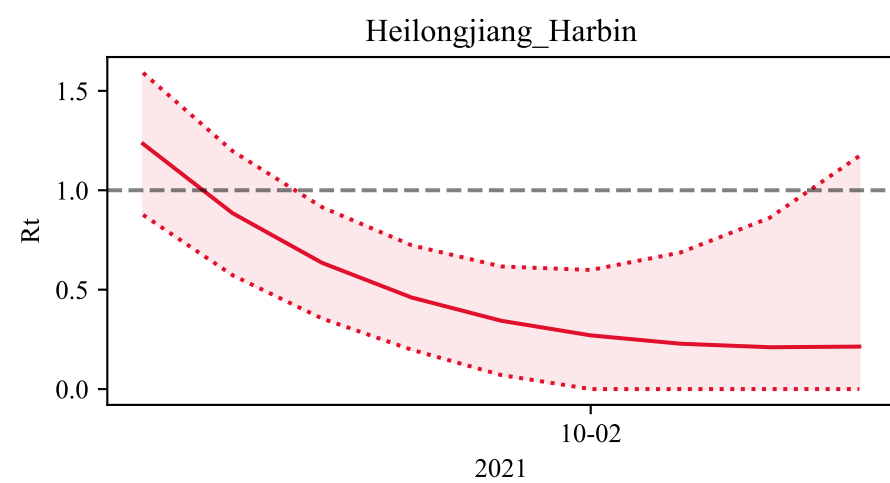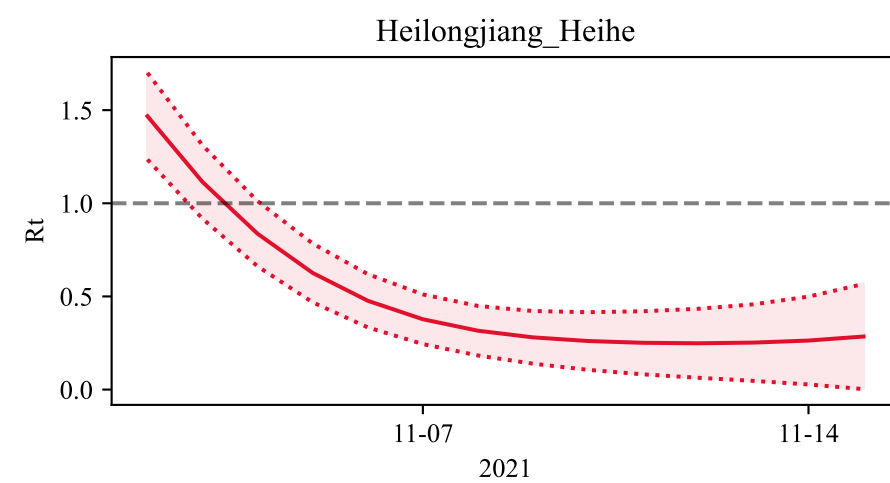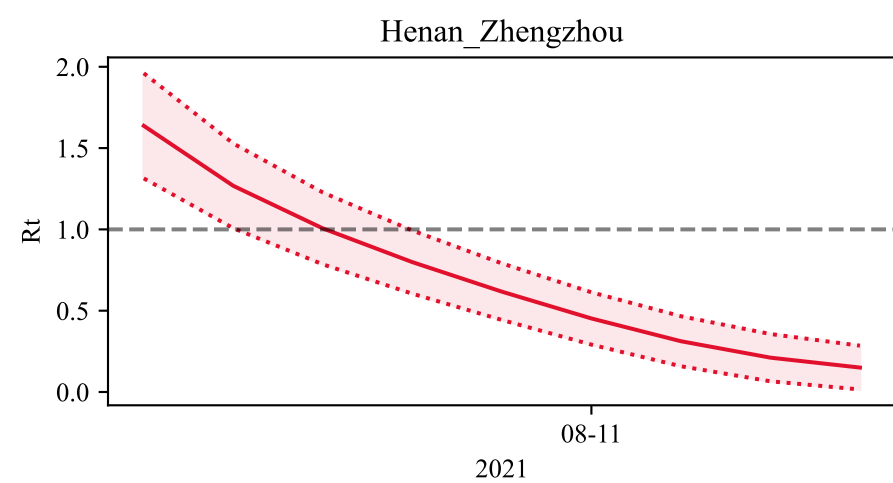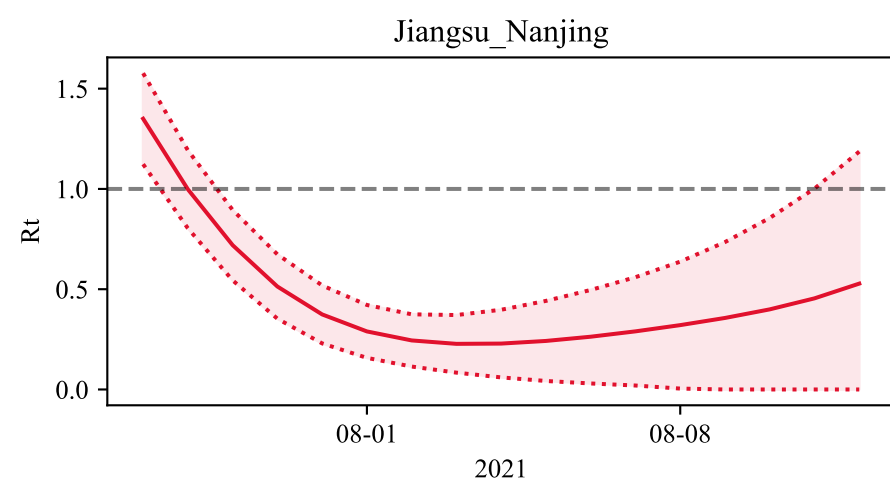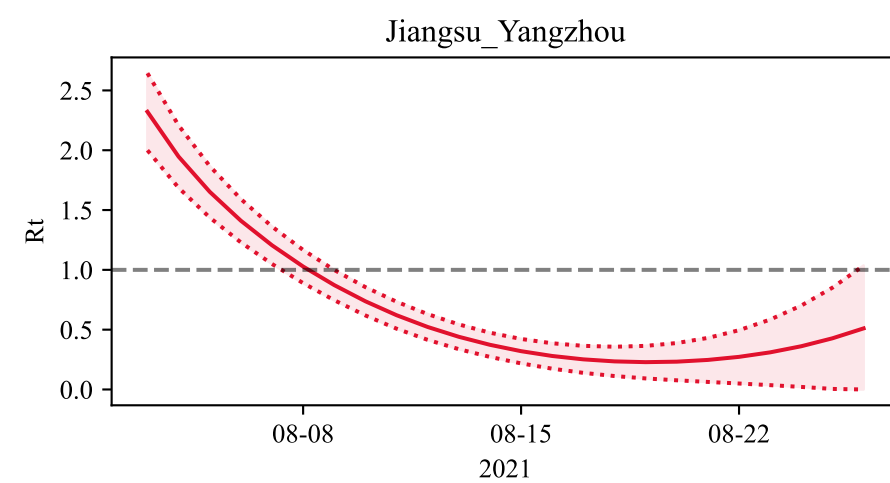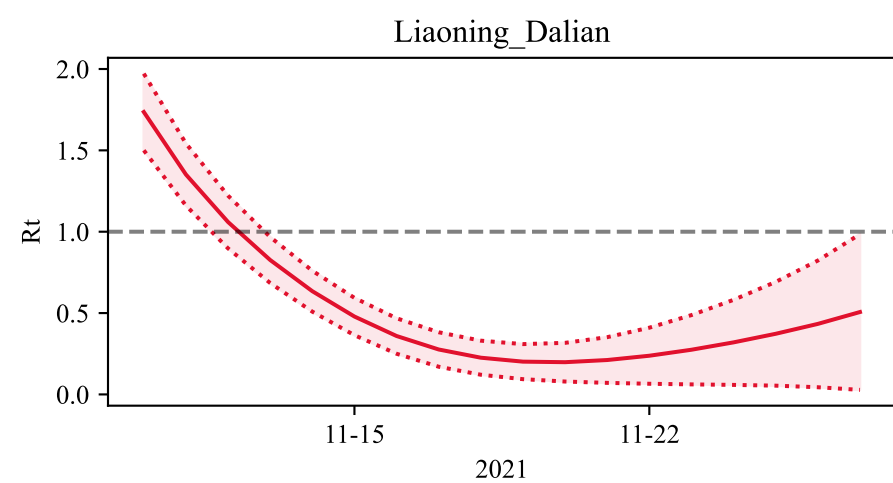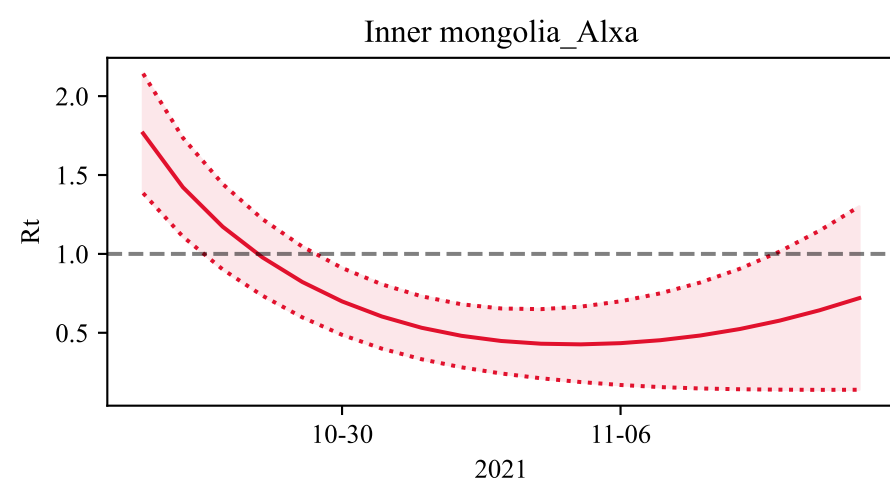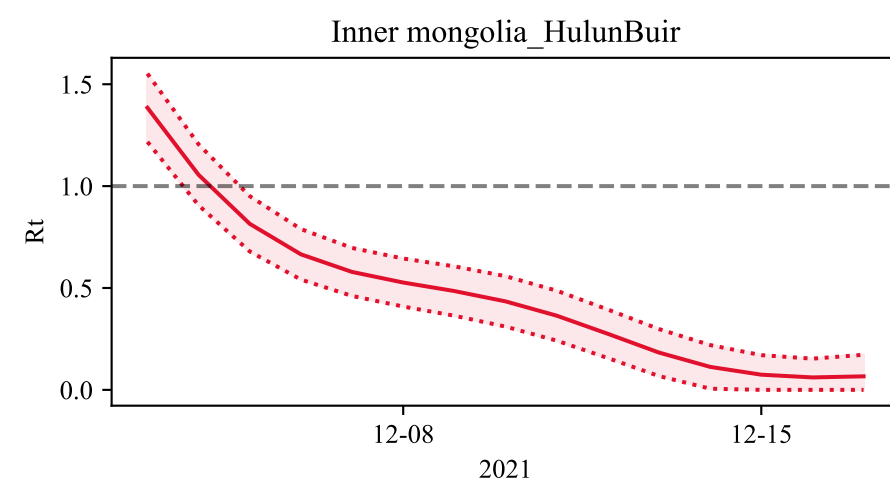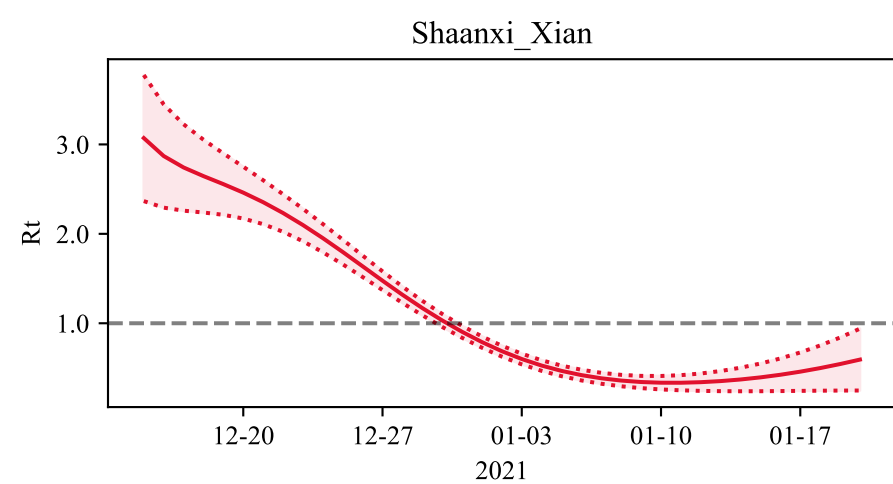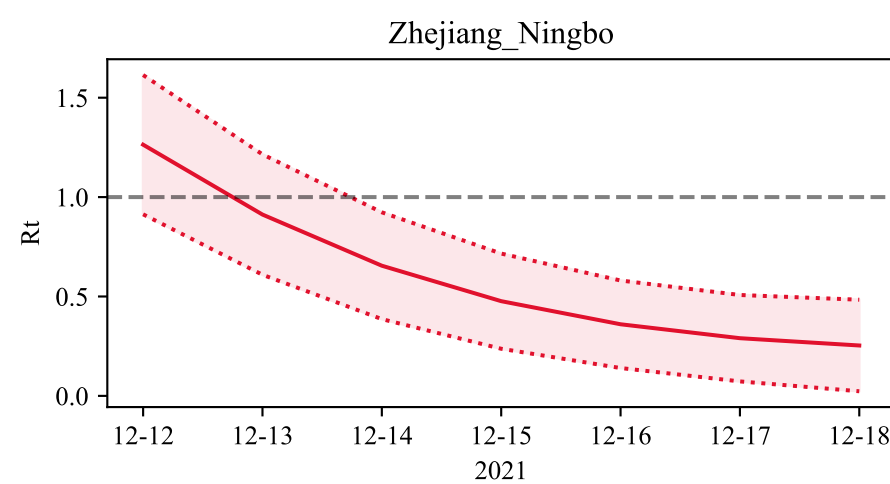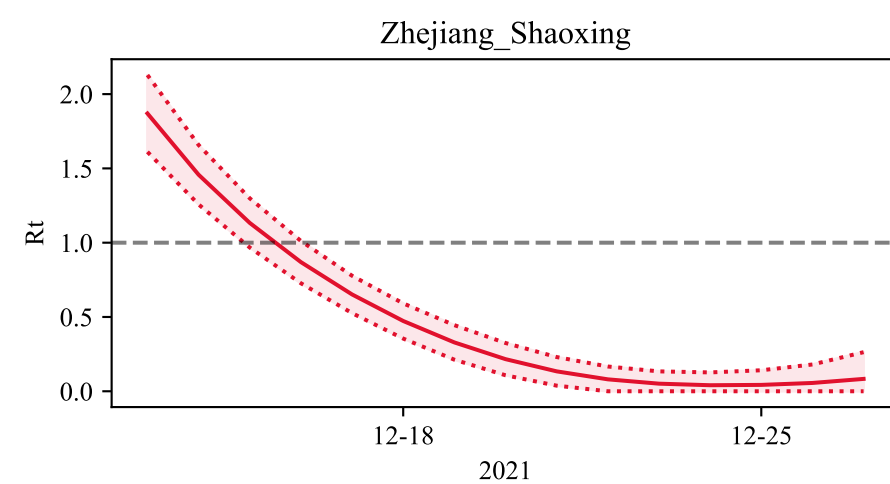

Supplement: Peng et al. supplementary material 9 — Peng et al. supplementary material [file S0950268824000360sup009.pdf]

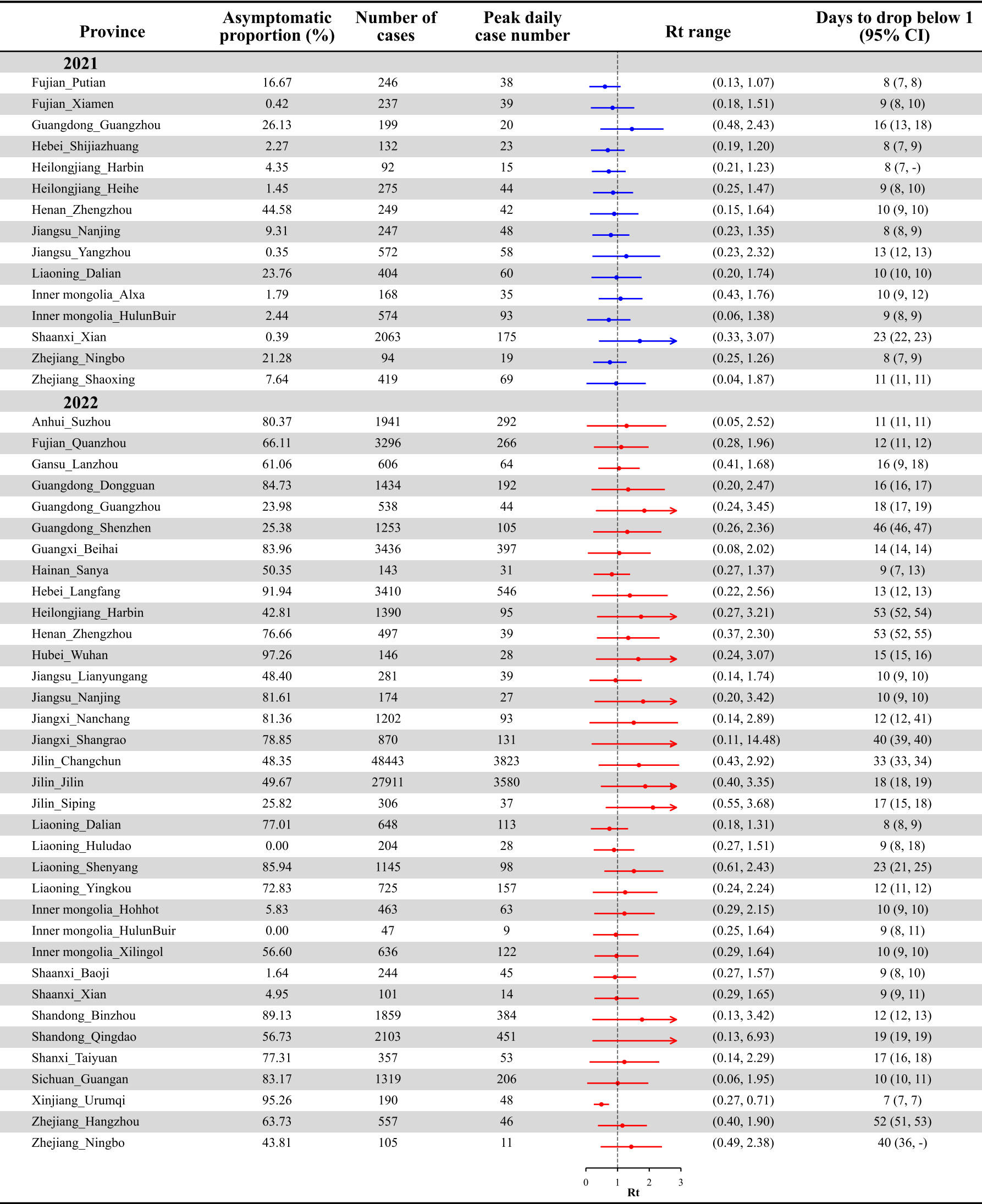

Supplement: Peng et al. supplementary material 10 — Peng et al. supplementary material [file S0950268824000360sup010.pdf]
